# Metacognition in AI-Supported Second Language Learning: A Systematic Review of Constructs, Measures, and Evidence (2016–2026)

**DOI:** 10.3390/jintelligence14100236

**Published:** 2026-10-01

**Authors:** Hong Yi, Qiang Chen, Zhuo Wang

**Affiliations:** 1 College of Foreign Languages, Hubei Minzu University, Enshi 445000, China; 2 Library of Yunnan University, Kunming 650091, China; 3 College of Educational Sciences, Qingdao University, Qingdao 266071, China

**Keywords:** metacognition, metacognitive accuracy, AI-supported language learning, generative artificial intelligence, systematic review

## Abstract

Metacognition has informed research on second and foreign language (L2) learning for three decades, while AI tools have supported such learning for nearly as long. Yet, no review has examined how the field conceptualises and measures metacognition under AI mediation. This systematic review identified 60 eligible studies published between 2016 and May 2026. Each study was coded on fourteen dimensions anchored in established taxonomies and appraised with the Mixed Methods Appraisal Tool. Only four eligible studies fall within the pre-ChatGPT portion of the review window, from January 2016 to November 2022; 56 were published during the subsequent three and a half years. Three findings emerge. First, measurement is narrowly concentrated: 98% of the 56 post-ChatGPT studies measured self-reported metacognitive regulation, and 66% relied on self-report alone. Only one study in the full corpus measured metacognitive accuracy. Five post-ChatGPT studies combined an AI-absent comparison with process-level data, and only one of these also measured judgement accuracy. Second, 41% of post-ChatGPT studies used tools that combined two or more functional orientations within a single interface, a configuration not represented by the field’s inherited four-category taxonomy of AI applications. Third, none of the 18 contrast-based studies assigned the regulatory operation to the system in place of the learner. In every design that specified the division of labour, the learner retained that operation; three designs did not specify it. The condition most in need of testing therefore remains untested. Reported findings skew positive, but correlational and qualitative designs account for a disproportionate share of that evidence. The review concludes with a research agenda centred on calibration and process data.

## 1. Introduction

Metacognition broadly refers to individuals’ knowledge, awareness, and regulation of their own cognitive processes. Since Flavell’s (1979) foundational formulation, the construct has commonly been understood as encompassing both metacognitive knowledge—what individuals know about themselves as learners, the nature and demands of a task, and the strategies available for accomplishing it—and metacognitive regulation, through which learners plan, monitor, and evaluate their cognitive activity (Schraw & Dennison, 1994; Veenman et al., 2006). Rather than simply possessing strategies, metacognitively competent learners are able to judge what a task requires, select appropriate strategies, monitor whether those strategies are working, and revise their approach when necessary. This capacity for higher-order oversight distinguishes metacognition from cognition itself: cognition enables learners to perform a task, whereas metacognition enables them to understand and regulate how they perform it (Veenman et al., 2006).

The importance of metacognition becomes particularly apparent when learning is complex, extended, or insufficiently structured. Successful learners do not merely invest cognitive effort; they allocate that effort strategically. They establish goals, identify gaps in understanding, monitor progress, evaluate the adequacy of their responses, and decide whether another strategy is required. Metacognition therefore provides a mechanism through which learners adapt their cognitive resources to changing task demands. Importantly, such regulation is not simply a correlate of general intellectual ability. Metacognitive skills make a distinctive contribution to learning and can themselves be developed through appropriate instruction (Veenman et al., 2006). Because these skills are teachable, instruction can improve learning by strengthening how learners acquire, use, and evaluate knowledge, not only by increasing what they know.

## 2. Literature Review

### 2.1. Metacognition in Second-Language Learning

Metacognition is especially consequential in second- and foreign-language (L2) learning because language learning requires learners to make repeated decisions under conditions of incomplete knowledge. Learners must determine, for example, whether they have understood a spoken passage sufficiently, what to do when comprehension fails, whether a written expression communicates their intended meaning, what vocabulary requires further attention, and whether feedback warrants revision. Accordingly, metacognition has been examined across listening, reading, writing, vocabulary learning, and broader language-learning strategy research. An early systematic synthesis by Raoofi et al. (2014), covering 33 empirical studies published between 1999 and 2013, concluded that metacognition generally contributed positively to second- and foreign-language learning while also identifying substantial variation associated with learner and contextual characteristics.

Listening provides one of the most extensively studied examples. Vandergrift et al. (2006) conceptualised successful L2 listening in terms of learners’ awareness and regulation of processes including planning and evaluation, problem solving, directed attention, mental translation, and person knowledge. Their Metacognitive Awareness Listening Questionnaire, developed with 966 language learners, subsequently became one of the most widely used measures of L2 listening metacognition. Research based on this tradition has consistently suggested that successful listening involves more than linguistic decoding: learners need to anticipate task demands, recognise comprehension breakdowns, redirect attention, test interpretations, and evaluate whether their strategies have been effective (Vandergrift et al., 2006; Vandergrift & Goh, 2012).

Comparable patterns have been observed in L2 writing. Writing requires learners to coordinate idea generation, linguistic formulation, organisation, monitoring, revision, and evaluation, making it particularly dependent on regulatory activity. F. Teng (2016), for example, found that embedded metacognitive instruction improved Chinese EFL learners’ writing performance and regulation of cognition, with benefits extending beyond the immediate instructional period. More recent work has similarly shown that metacognitive processes are intertwined with how learners interpret task demands, deploy writing strategies, respond to difficulties, and evaluate their developing texts. Rather than representing a single generalised ability, L2 writing metacognition appears to involve interrelated forms of knowledge, experience, monitoring, and regulation whose intensity is moderated by factors including time, individual learner profiles, intrinsic motivation, external feedback, and linguistic competence (Z. Li et al., 2026).

Longitudinal research further suggests that metacognition should not be treated simply as a stable attribute that some learners possess more strongly than others. Following 2126 secondary-school EFL learners across three measurement occasions, Cai (2024) identified distinct developmental patterns in metacognitive strategy use, demonstrating that learners’ regulatory practices can change over time and in relation to their language development. Contemporary reviews likewise portray L2 metacognition as both consequential and malleable: it is associated with successful language learning, but its manifestation depends on the interaction among learners, tasks, instructional environments, and available learning resources (Z. Li et al., 2026; Raoofi et al., 2014). Collectively, this literature positions metacognition not as an ancillary learning strategy but as an important mechanism through which language learners exercise control over their own learning.

### 2.2. AI and the Changing Conditions of Language Learning

The conditions under which such control is exercised have changed substantially with the growing incorporation of artificial intelligence (AI) into language learning. Long before the emergence of ChatGPT, AI-supported language technologies included intelligent tutoring systems, automated writing evaluation, machine translation, speech-recognition systems, adaptive learning environments, recommender systems, and conversational agents. Such technologies have been used to diagnose errors, provide individualised feedback, assess proficiency, recommend learning materials, facilitate interaction, and adapt instruction to learner performance (Chang & Sun, 2024; S. Qiao et al., 2025). Reviews of earlier AI-supported language learning generally suggest beneficial effects on language performance, while also documenting considerable heterogeneity in technological design, instructional implementation, research quality, and outcome measurement (S. Qiao et al., 2025).

The emergence of generative artificial intelligence (GenAI), particularly large language model-based systems, has expanded these possibilities considerably. Systems such as ChatGPT, Gemini, Claude, Microsoft Copilot, and DeepSeek can sustain open-ended dialogue and generate, reformulate, explain, translate, summarise, evaluate, and respond to language in real time. Unlike many earlier AI technologies designed to perform a relatively bounded instructional function, a single GenAI system may simultaneously serve as a conversational partner, writing assistant, translator, feedback provider, vocabulary resource, idea generator, evaluator, or on-demand tutor. Learners can request alternative explanations, ask follow-up questions, simulate conversations, generate examples, compare expressions, obtain feedback on drafts, or repeatedly revise prompts and responses. This multifunctionality helps explain the rapid expansion of GenAI research in language education: B. Li et al. (2025), for example, identified 144 empirical studies published in only the first two full years of widespread GenAI use, 2023–2024.

Emerging empirical studies document substantial potential benefits. In EFL writing, learners have reported using GenAI and related AI tools for planning, idea development, language problem solving, feedback, editing, and revision rather than for a single uniform purpose (M. F. Teng, 2025b; Y. Wang, 2024). Y. Wang’s (2024) study of academic EFL writing, for example, showed that students integrated machine translation and GenAI tools into multiple phases of their self-regulated writing processes. In reading, Pan et al. (2025) found that GenAI-empowered interactive support significantly strengthened university EFL learners’ self-regulated reading-strategy use and reading engagement. GenAI can also lower barriers to obtaining individualised linguistic assistance by providing immediate explanations, examples, reformulations, and feedback that would otherwise require access to a teacher, tutor, or more proficient interlocutor (B. Li et al., 2025; S. Qiao et al., 2025).

These affordances, however, are accompanied by substantial risks. At the output level, GenAI can generate inaccurate, fabricated, culturally or pragmatically inappropriate, excessively generic, or contextually unsuitable responses. At the learning-process level, a more fundamental concern arises: increasingly capable AI can alter who performs the cognitive work of learning. Activities that once required learners to retrieve vocabulary, formulate sentences, develop arguments, diagnose errors, compare alternatives, evaluate feedback, or reformulate an unsuccessful response can now be partially or almost completely delegated to an external system. Consequently, improvement in an AI-assisted product cannot automatically be equated with improvement in the learner. A learner may produce a more fluent essay, answer a question more accurately, or complete a task more efficiently while engaging less fully with the cognitive processes through which durable knowledge and independent competence develop.

This distinction is especially consequential in language learning. Retrieval, noticing gaps in knowledge, struggling to formulate an expression, detecting errors, comparing alternatives, and reformulating unsuccessful language are not simply inefficiencies that technology should eliminate; they may themselves constitute opportunities for learning. AI therefore creates a tension that was much less pronounced with many earlier educational technologies. The same assistance that makes language learning more accessible and efficient may also reduce the need for learners to engage in some of the processes through which language competence and learning autonomy are developed.

### 2.3. Three Loci for the Regulatory Operation

The metacognitive consequences of AI depend on the regulatory operations it performs rather than on the technology alone. A more tractable question follows from the meta-level–object-level architecture proposed by Nelson and Narens (1990), in which cognition about a task proceeds at an object level while monitoring and control operate at a meta level that receives information from that level and issues commands to it. When a second agent enters the task, the analytic question becomes which of those meta-level operations the agent performs and which remain with the learner.

Three loci can be distinguished. AI may prompt a regulatory operation while leaving its execution to the learner (scaffold); it may perform part of the operation while the learner retains judgement over the result (transformation); or it may execute the operation and deliver its product (substitution). The relevant question is which cognitive and metacognitive responsibilities remain with the learner once AI enters the task.

In the first locus, AI can function as a powerful metacognitive scaffold. Learners can use an AI system to formulate learning goals, decompose a difficult task, consider alternative strategies, identify areas of weakness, obtain feedback, generate reflective questions, compare an initial response with a revised one, or explain why one solution may be preferable to another. Properly structured, such interaction can externalise normally implicit processes of planning, monitoring, and evaluation and make them more accessible to learners. The regulatory operation itself, however, is still carried out by the learner: the system supplies an occasion for monitoring, not a monitoring judgement.

Transformation, the second locus, is the least well conceptualised and has become routine with generative systems. In this configuration, AI performs part of the meta-level operation and returns the result for the learner to evaluate. A system that diagnoses which features of a draft are weak has carried out the comparison against a standard that the learner would otherwise have had to make, yet what follows from that diagnosis remains the learner’s decision.

What learners then do with the returned diagnosis is neither uniform nor assured. Zhan and Yan (2025), who observed sixteen undergraduates working with ChatGPT feedback on IELTS writing tasks, found that the students used more cognitive than metacognitive strategies, differed in how much they trusted the feedback, and engaged only superficially when requesting feedback, interacting with the system, and revising. Ma et al. (2026), following six Chinese EFL writers through revision, found them more receptive to local than to global feedback while setting goals, retrieving prior knowledge and evaluating as they went. The division of regulatory labour is genuinely altered rather than merely supported, but the learner remains in the loop with a judgement to make.

The boundary between scaffolding and transformation is conceptually consequential, as discussed further in Section 4.5. Whether supplying diagnostic information already constitutes performing a meta-level operation, or merely furnishes the evidence on which the learner performs it, cannot be settled by observing the system alone; it depends on what the learner would otherwise have had to generate. We retain the distinction because the two cases imply different consequences for what the learner practises, while acknowledging that primary studies rarely report enough about the alternative available to the learner to place a deployment on one side of the boundary with confidence.

Substitution completes the sequence. If learners routinely ask AI to determine what matters, decide how a task should be approached, identify errors, judge whether an answer is adequate, decide what requires revision, and then perform that revision, they may progressively outsource metacognitive regulation rather than exercise it. Under such conditions, AI moves from supporting the learner’s regulatory loop to occupying parts of that loop. The distinction is theoretically important. Asking an AI system for feedback and then evaluating its validity requires monitoring and judgement; accepting the same feedback uncritically does not. Asking AI to suggest several possible strategies and deciding among them may stimulate metacognition; asking it to choose and execute the strategy may circumvent the learner’s regulatory activity.

Because the locus depends on the learner as much as on the system, seemingly similar AI use can produce different learning processes. A learner with stronger monitoring and evaluation skills may question an implausible suggestion, compare it against prior knowledge, request clarification, and decide selectively whether to incorporate it. Another learner may accept the same output because it appears linguistically polished and authoritative. In the first case, AI becomes an object of metacognitive scrutiny; in the second, it may become a substitute for that scrutiny. The technology is the same, but the distribution of epistemic and regulatory responsibility is different.

### 2.4. Evidence for Each Locus and the Conditions That Qualify It

Empirical support for the scaffolding locus is the most developed of the three. Pan et al. (2025), for example, found that GenAI-empowered interactive support enhanced EFL learners’ self-regulated reading-strategy use as well as their engagement. Y. Wang (2024) similarly documented EFL writers’ strategic integration of machine translation and GenAI at different stages of academic writing, including planning, problem solving, and revision. Research focusing more directly on metacognition also suggests that learners can engage reflectively with GenAI. M. F. Teng (2025a), examining EFL learners’ use of ChatGPT for writing feedback, found that learners’ metacognitive awareness was closely implicated in how they perceived and experienced AI-generated feedback. Abdelhalim (2024), in research on EFL undergraduates’ use of ChatGPT to develop research competency, likewise showed that learners differed in how strategically and critically they incorporated the system into their work.

Evidence outside L2 learning strengthens this interpretation. X. Xu et al. (2025), for instance, quasi-experimentally examined the incorporation of explicit metacognitive support into a GenAI learning environment. In a four-week study with 68 undergraduates, learners receiving explicit metacognitive prompts developed stronger self-regulated learning, particularly in task strategy and self-evaluation, although the intervention produced no corresponding significant between-group advantage in academic performance. What such findings isolate is not the presence of a capable model but an interaction structured so as to preserve and activate the learner’s own regulatory processes.

Available findings are consequently mixed rather than uniformly favourable. Some studies show greater strategic engagement, reflection, and self-regulation in AI-supported environments (Pan et al., 2025; Y. Wang, 2024; X. Xu et al., 2025), whereas others indicate that the benefits of GenAI depend heavily on learners’ capacity to interrogate, evaluate, and selectively use its output (Abdelhalim, 2024; M. F. Teng, 2025a). M. F. Teng’s (2025a) findings are particularly instructive in this regard because metacognitive awareness was not merely an outcome potentially affected by ChatGPT; it also shaped how learners engaged with ChatGPT feedback. AI may therefore influence metacognition, while learners’ existing metacognition simultaneously determines what they do with AI.

Experimental evidence also raises concerns that improvements in AI-assisted performance may coexist with weaker engagement in the processes underlying durable learning. Y. Fan et al. (2025), in a randomised study comparing learners supported by ChatGPT, a human expert, writing analytics, or no additional support, found that the ChatGPT group achieved greater improvement in immediate essay performance but did not demonstrate corresponding advantages in knowledge gain or transfer. The groups also differed significantly in the frequency and sequencing of their self-regulated learning processes. Fan et al. used the term metacognitive laziness to describe the possibility that highly capable GenAI support may enable learners to achieve better immediate products while reducing the regulatory effort normally required to produce them. Although their study was not conducted specifically with L2 learners, the mechanism is highly relevant to language learning because GenAI can now assume an unusually large share of linguistic formulation, diagnosis, and revision.

What separates the two outcomes appears to be instructional rather than technological. Synthesising 67 empirical studies of ChatGPT and university students’ thinking, C. Li et al. (2026) found that its cognitive effects were contingent on pedagogical framing: embedded in inquiry-oriented, scaffolded designs, ChatGPT supported metacognitive regulation and argumentative reasoning, whereas unstructured use was associated with cognitive offloading. This distinction is critical. Traditional cognitive offloading might involve using a dictionary instead of remembering a word or a calculator instead of performing arithmetic mentally. GenAI can go further: it can decide what the problem is, recommend what the learner should do, evaluate whether the resulting response is adequate, and formulate the improved response. The object of delegation can therefore shift from cognition to the regulation of cognition itself.

This literature identifies three features of AI deployment as candidate conditions; the review therefore specified and coded them before examining their association with outcomes. The first is whether explicit metacognitive scaffolding was embedded in the intervention: F. Teng (2016) found that embedded metacognitive instruction improved Chinese EFL learners’ writing and their regulation of cognition, with effects persisting beyond the instructional period, and C. Li et al. (2026) locate the same contrast at the level of pedagogical framing. The second is the length of the AI-exposure period, since Cai (2024) demonstrated across three annual waves with 2126 secondary-school learners that metacognitive strategy use fluctuates rather than holding constant, so a brief exposure may capture a transient state rather than a developed capability.

The third candidate condition is whether use was teacher-guided or learner-directed, because unstructured use is the configuration C. Li et al. (2026) associate with offloading and X. Xu et al. (2025) contrast with explicit prompting. Whether a study states a learning theory is deliberately not treated as a condition of this kind: it is a property of the report rather than of the learner’s task environment, and it is reported in this paper as a descriptive characteristic of the literature.

### 2.5. Calibration and the Offline–Online Distinction

A further problem follows from these possibilities but has received comparatively little attention in AI-supported L2 research: metacognitive accuracy or calibration. Metacognition concerns not only whether learners plan, monitor, and evaluate, but also whether their judgements about their own knowledge and performance correspond to what they actually know and can do. A learner can be highly confident yet inaccurate, or uncertain yet competent. Thus, an increase in perceived understanding, confidence, or self-assessed proficiency does not necessarily indicate improved metacognitive functioning if the learner’s judgements become less aligned with objective performance.

The absence of calibration from AI-supported L2 research is consequential because established methods already exist for measuring it. Nelson and Narens (1990) formalised monitoring as a family of judgements—of learning, of confidence, of feeling of knowing—whose accuracy can be quantified against subsequent performance, and Nelson (1984) compared the measures of accuracy that result. Schraw (2009) sets out five distinct measures of metacognitive monitoring, which is itself a warning that the choice of index is not neutral. Bjork et al. (2013) show why the issue is not merely technical: learners’ ongoing assessments of their own learning are shaped by current performance and by the subjective sense of fluency, which is what leaves beliefs about one’s own learning open to illusion.

GenAI may make this distinction particularly consequential. When learners work with systems capable of generating fluent language and polished products, the performance visible during an AI-supported task represents the performance of a human–AI system, not necessarily the learner’s independent competence. A learner may therefore experience successful task completion and infer that the underlying knowledge or skill has been mastered, even when the performance cannot be reproduced without AI assistance. The fluency that shapes such a judgement is in this case supplied by the system rather than generated by the learner. Y. Fan et al.’s (2025) finding of improved immediate products without corresponding advantages in knowledge gain or transfer illustrates why this possibility warrants attention. The important metacognitive question is not merely whether learners feel more capable after using AI, but whether what they believe they can do corresponds to what they can actually do independently.

Answering this question requires researchers to select an appropriate measurement modality before collecting data. Veenman et al. (2006) distinguish offline measures, obtained before or after a task and dependent on the learner’s recollection and self-perception, from online measures, registered during performance as regulation occurs; the two families converge only weakly, because the first captures what learners believe about their strategies and the second what they do.

The most established L2 instruments are offline by design: both the Metacognitive Awareness Listening Questionnaire (Vandergrift et al., 2006) and the Metacognitive Awareness of Reading Strategies Inventory (Mokhtari & Reichard, 2002) ask learners to characterise habitual strategy use, whereas think-aloud protocols, trace and log data, and performance-linked judgement accuracy observe the operation itself. AI mediation widens the gap, because a learner’s report now covers an activity performed jointly with a system whose contribution is not separately available for introspection. Measurement modality therefore determines which aspects of metacognition the literature can identify; accordingly, this review codes modality explicitly (Section 3.5).

Taken together, the emerging evidence is better characterised as conditional and mixed than as consistently positive or negative. AI can scaffold planning, monitoring, evaluation, and strategic adaptation when learners remain cognitively and epistemically engaged with its output; it can also assume these functions when learners delegate judgement to the system. Metacognition may consequently operate simultaneously as an outcome of AI-supported learning, a resource that determines the quality of learners’ AI use, and a process through which human and artificial agents divide regulatory responsibility (Abdelhalim, 2024; Pan et al., 2025; M. F. Teng, 2025a; Y. Wang, 2024; X. Xu et al., 2025). These competing possibilities make metacognition a particularly important lens for understanding why AI-supported performance does not always translate into independent learning.

### 2.6. Contribution and Gaps of Existing Reviews

A growing review literature has approached this intersection from several directions, yet none has made metacognition proper the organising construct for synthesising AI-mediated L2 learning. Six recent syntheses are particularly relevant.

Chang and Sun (2024) systematically reviewed AI-supported self-regulated language learning between 2000 and 2022 through the lens of Zimmerman’s (2000) cyclical model. Their review established an important foundation by showing how AI technologies can participate in the forethought, performance, and self-reflection phases of language learners’ self-regulation. It also foregrounded the potential metacognitive significance of AI as a learning partner or “mind tool.” However, self-regulated learning is conceptually broader than metacognition: SRL incorporates motivational, behavioural, and environmental regulation alongside metacognitive knowledge and regulation (Panadero, 2017). Consequently, a synthesis organised around SRL cannot determine with sufficient precision which metacognitive components are being supported, altered, or displaced. Its search window also ended in 2022, immediately before the rapid proliferation of empirical studies involving ChatGPT and subsequent GenAI systems.

Z. Zhang et al. (2025) moved considerably closer to the present problem in their systematic scoping review of empirical research at the intersection of AI, SRL, and L2 education. Particularly useful is their identification of three ways SRL has been positioned in AI-mediated language-learning research: as an outcome of AI use, as a framework guiding AI-supported instructional design, and as a strategic process enacted by learners while interacting with AI. The same tripartite distinction can be productively transposed to metacognition. Metacognition may be changed by AI, deliberately scaffolded through AI-based instructional designs, or enacted by learners as they interrogate and evaluate AI output. Nevertheless, Z. Zhang et al. (2025) retain SRL in its full breadth as the focal construct. Their synthesis therefore does not isolate metacognitive knowledge, monitoring, evaluation, or calibration from the motivational and behavioural components of self-regulation.

Nguyen et al. (2026) address another important dimension: the distinction between earlier AI and generative AI. Their review compares AI-supported SRL studies published between 2015 and 2025 using the COPES architecture and shows how GenAI differs from earlier systems in the forms of adaptive, dialogic, and generative support it can provide. This generational distinction is particularly important for metacognition because GenAI can participate in planning, diagnosis, evaluation, and decision making to an extent that many earlier, function-specific AI systems could not. However, Nguyen et al.’s synthesis spans educational domains rather than focusing specifically on second- and foreign-language learning, and metacognition remains embedded within the broader architecture of SRL rather than serving as the focal construct. It also does not foreground calibration—the accuracy of learners’ judgements about their own knowledge and competence—as a distinct analytical problem.

The complementary limitation appears in broad reviews of AI-supported language learning. Y. Wang et al. (2026) traced two decades of AI in language learning across 272 empirical studies published between 2005 and 2024, providing a valuable historical picture of technological and research development across the field. Their review captures the transition from earlier specialised systems toward increasingly conversational and generative AI. Yet its purpose is field-level mapping: metacognition, when present, represents one process or outcome among many rather than the central unit of analysis.

B. Li et al. (2025) offer the corresponding picture of the early years of generative AI. Their systematic review synthesised 144 empirical studies published from 2023 to 2024, documenting the extraordinary speed with which GenAI became integrated into language learning and teaching. The review maps applications, research trends, learner and teacher experiences, outcomes, and emerging challenges across the rapidly developing field. Its breadth is valuable for establishing what GenAI has been used to do in language education, but the same breadth means that metacognition is not isolated as an analytical construct. It therefore cannot establish whether the new generation of AI technologies systematically changes learners’ planning, monitoring, evaluation, knowledge of cognition, or judgement accuracy.

Finally, S. Qiao et al. (2025) systematically reviewed the design, theoretical foundations, implementation, and impact of AI technologies for language learning, focusing on experimental and quasi-experimental evidence published through 2023. Their review provides an important picture of the largely pre-generative or transitional AI landscape and highlights both the potential of AI-supported language learning and limitations in the strength and consistency of the available evidence. Yet metacognition is again not the organising construct, and the temporal scope largely precedes the period in which open-ended GenAI systems became everyday learning tools capable of taking over substantial portions of learners’ cognitive and regulatory work.

Viewed together, these six syntheses reveal a construct–domain–generation gap. Reviews located at the intersection of AI and L2 self-regulation retain SRL as the overarching construct and therefore do not isolate metacognition (Chang & Sun, 2024; Z. Zhang et al., 2025). The review that most directly compares earlier AI and generative AI retains the broader SRL construct and extends across educational domains rather than concentrating on L2 learning (Nguyen et al., 2026). Conversely, the major reviews that map AI or GenAI in language learning provide the necessary domain specificity but not the necessary construct specificity (B. Li et al., 2025; S. Qiao et al., 2025; Y. Wang et al., 2026). Together, these limitations leave metacognition under AI mediation in L2 learning without a construct-specific, domain-specific, and generation-sensitive synthesis.

A second gap cuts across all three strands. Existing syntheses concentrate overwhelmingly on whether learners use metacognitive or self-regulatory strategies and whether AI supports those strategies; they give much less attention to whether learners’ metacognitive judgements are accurate. This omission matters because the post-ChatGPT era changes the relationship between observable task performance and underlying learner competence. When AI contributes directly to a successful product, learners may have fewer cues for determining which part of that success reflects their own knowledge. Metacognitive calibration becomes especially important when learners and AI jointly produce task performance. Yet no existing synthesis has systematically examined whether AI-mediated L2 learning improves, preserves, or distorts the correspondence between learners’ perceived and demonstrated competence.

A focused synthesis is warranted because AI can intervene directly in the regulatory processes through which learners ordinarily control cognition, not merely because the two terms have not previously been reviewed together. AI can now participate in the same regulatory processes by which learners would ordinarily control their own cognition. It may scaffold those processes, transform how they are enacted, or partially substitute for them; at the same time, successful AI-supported performance may change learners’ judgements about what they themselves know and can do. Understanding these possibilities requires metacognition to be treated as the focal construct rather than one component of a larger SRL framework.

The present review addresses this gap by systematically synthesising empirical research specifically concerned with AI and metacognition in second- and foreign-language learning across both earlier and generative AI technologies. In contrast with broader SRL reviews, it differentiates metacognitive knowledge, regulatory processes, and—where evidence permits—metacognitive accuracy. In contrast with field-wide reviews of AI in language education, it places learners’ regulation and evaluation of their own cognition at the centre of analysis. Finally, by comparing evidence across the neural-AI and post-ChatGPT eras, it examines whether the increasing capacity of AI to generate, diagnose, evaluate, and revise language has changed the relationship between technology and learner metacognition.

Conceptually, the review examines whether AI functions as a metacognitive scaffold, a substitute for learners’ regulation, or a source of metacognitive miscalibration, rather than reducing its role to a positive or negative effect. This framework makes it possible to distinguish situations in which AI prompts learners to plan, monitor, question, and evaluate from those in which it performs these activities for them, while also considering whether AI-supported success leaves learners with an accurate understanding of their independent competence. Such distinctions may help explain why existing empirical findings are mixed and why superficially similar forms of AI use can produce substantially different learning processes.

Accordingly, the review addresses the following research questions:

RQ1. What are the characteristics and developmental patterns of empirical research on AI and metacognition in second- and foreign-language learning, particularly in relation to learner populations, language-learning domains, AI technologies, pre-generative versus generative AI, research designs, and approaches to conceptualising and measuring metacognition?
RQ2. What does existing empirical evidence indicate about the relationships between AI use and different dimensions of L2 learners’ metacognition, including metacognitive knowledge, planning, monitoring, evaluation, strategy regulation, and metacognitive calibration?
RQ3. How do study designs distribute regulatory responsibility between learner and system, and which learner-, task-, technology-, and instructional-level conditions accompany positive metacognitive outcomes?

## 3. Methodology

### 3.1. Design

This systematic review examined full-text studies that substantively measured learner metacognition in AI-mediated second or foreign language (L2) learning. The review followed the PRISMA 2020 statement (Page et al., 2021). A protocol was written prospectively, before screening and coding began, and is provided with a dated amendment log as Supplementary File S2. The review was not registered and no equivalent register was available for this topic.

### 3.2. Data Sources and Search Strategy

**Figure 1:**
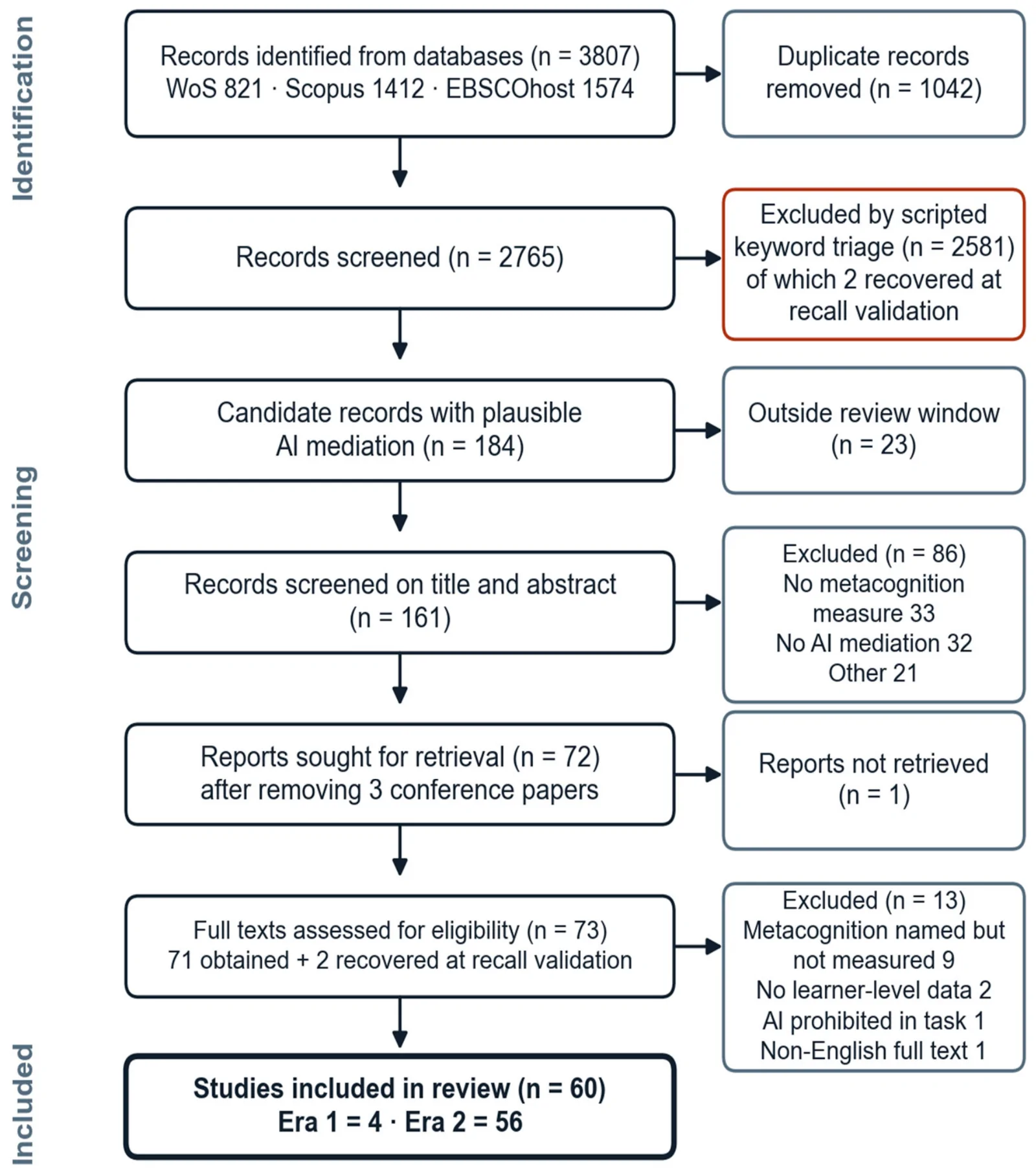
PRISMA 2020 flow diagram.

Searches were executed in June 2026 across six databases on three platforms: Web of Science Collection; Scopus; and, on EBSCOhost, Academic Search Complete, ERIC, APA PsycINFO, and Education Source (searched simultaneously). An update search was conducted on 7 August 2026 as a CrossRef-based supplementary search; the database searches themselves were not rerun on that date. The PRISMA counts and final corpus derive from this update. Search strings combined a metacognition module (metacogniti*, meta-cogni*, “meta cognit*”) with an L2 module (L2, “second language”, “foreign language”, EFL, ESL, TESOL) in topic or title–abstract–keyword fields. In Scopus, on EBSCOhost, and in Web of Science, these two modules were combined with an empirical filter (finding OR result OR experiment* OR control OR intervent*), drawn from Guan et al. (2024), applied to the abstract field in Web of Science and on EBSCOhost and to title–abstract–keyword fields in Scopus; on EBSCOhost, the two substantive modules were applied to subject headings. No technology or AI terms were included in the search strings. Pilot searches showed that AI vocabulary varied across the review window (e.g., “ChatGPT” was absent from controlled vocabularies); technological mediation was therefore assessed during screening rather than retrieval to favour recall over precision. Searches yielded 3807 records (Web of Science = 821; Scopus = 1412; EBSCOhost = 1574). The screening process is displayed in Figure 1.

### 3.3. Inclusion and Exclusion Criteria

Studies were eligible on four criteria. First, they involved learners of a second or foreign language at any educational level. Second, they featured AI mediation of the learning task. AI mediation was defined as an artificial-intelligence application standing between the learner and the language task: intelligent tutoring, automated writing evaluation, automatic speech recognition, adaptive systems, machine translation, dialogue agents, or generative large-language-model tools. Technologies used solely as research instruments, such as eye-tracking, did not qualify. Third, they substantively measured learner metacognition in any of four facets: metacognitive knowledge (Flavell, 1979; Wenden, 1998), metacognitive regulation (Brown, 1987; Schraw & Dennison, 1994), metacognitive experiences (Efklides, 2011), or metacognitive accuracy and calibration (Nelson & Narens, 1990). Fourth, they reported empirical data from human participants. Substantive measurement required either a recognised metacognitive instrument or subscale, or analytic categories substantively equivalent to metacognitive knowledge or regulation with separately reportable results. Generic self-regulated-learning strategy questionnaires with no metacognitive conceptualisation anywhere in the article did not qualify. Following Dinsmore et al. (2008), equivalence was judged on actual operationalisation rather than terminology, in both directions: studies were not excluded merely for avoiding the term metacognition, nor included merely for using it. Studies were dated by earliest public availability rather than by issue year, and era assignment follows that date; where the two differ, the reference list gives the issue year while the coding tables in Supplementary Files S4 and S5 give both.

The review window was 1 January 2016 to 31 May 2026, dated by earliest public availability (online-first) rather than by issue year; sixteen candidate records changed era assignment under this rule; reference entries follow the version of record, whose issue year is in some cases later. The window opens at the neural turn in language technology—neural machine translation and neural speech recognition reached mainstream products in 2016—and the two eras were divided at 30 November 2022, the public release of ChatGPT: a neural-AI era (Era 1) and a post-ChatGPT era (Era 2). Conference papers, non-peer-reviewed articles, non-English journal articles, and papers without obtainable full text were excluded.

### 3.4. Study Selection

After de-duplication (DOI-first, with normalised-title fallback; 1042 records removed), 2765 records proceeded to screening. A scripted keyword triage against the three eligibility modules identified 184 candidate records with plausible AI mediation, of which 161 fell within the review window. Title-and-abstract screening of these 161 records excluded 86 (principally: no substantive metacognition measurement, *k* = 33; no AI mediation, *k* = 32) and retained 75 for full-text assessment; three conference papers were then removed under the document-type rule. Of the 72 target articles, 71 full texts were obtained (98.6%); one (closed-access, no open-access version, author request unanswered) could not be retrieved. At full-text assessment, 13 articles were excluded. In nine, metacognition was named but not substantively measured: a label-only mention, a generic SRL composite with no metacognitive conceptualisation, or a construct anchored in a different theoretical tradition. In a further two, no learner-level metacognition was available at all, because the participants were teachers or the unit of analysis was the feedback comment. In one study, AI was prohibited in the target task. One further article proved, on full-text inspection, to be published in Spanish beneath an English title and abstract, violating the language criterion; a full-text language audit of all 71 retrieved articles found no further cases. The validation of the triage reported below recovered two further eligible studies that the script had wrongly excluded; both were added at full-text assessment. The final corpus comprised 60 studies. The 60 included studies are listed in Appendix B (Abdelhalim, 2024; Aldamen et al., 2026; Ali, 2026; Al-Othman, 2024; Amani & Bisriyah, 2025; Barrios-Beltran, 2025; J. Chen & Alibakhshi, 2026; M.-R. A. Chen, 2024; Y. Chen & Shim, 2026; Z. Chen & Goh, 2026; Choi, 2026; Cong-Lem, 2025; Dai, 2026; Dhanapal et al., 2026; Dillon, 2024; Du, 2025; Ebrahimi & Chowdhury, 2025; K. Fan & Gao, 2026; García Botero et al., 2021; Geng & Yamada, 2025; C. Hao et al., 2025; L. Hao et al., 2026; H. Hong et al., 2025; Hsu et al., 2024; Hu & Yordchim, 2025; Kessler, 2023; Lai et al., 2026; Lee, 2024; K. Li et al., 2026; Lin et al., 2026; Liu & Li, 2025; Lu & Zeng, 2026; Lukešová & Jennings, 2026; Luo & Yusuf, 2026; Ma et al., 2026; Namaziandost, 2025; H. Qiao & Zhao, 2023; Rababah et al., 2026; Rogti & Ouarniki, 2026; Schenck et al., 2026; Shen & Tao, 2025; Tekir, 2026; M. F. Teng, 2025a, 2025b, 2026; M. F. Teng & Shen, 2025; M. Wang & Zhang, 2026; P. Wang et al., 2025; S. Wang et al., 2026; X. Wang et al., 2025; Williyan & Fitriati, 2025; Q. Xu & Wang, 2025; Yan & Zhang, 2024; Yang & Wang, 2020; Yao & Fan, 2026; Yifan et al., 2025; Yin, 2025; Zablotska et al., 2026; A. Zhang & Jiang, 2026; J. Zhang & Zhang, 2024).

The recall of the scripted triage was evaluated through an additional validation procedure. All 2581 records excluded by the script were re-screened against a deliberately over-inclusive lexicon that strictly contained the original one; on the 184 candidates the original procedure had retained, the expanded lexicon achieved complete recall. Every excluded record the lexicon flagged as combining AI, metacognition and L2 vocabulary within the window was then screened manually as a census (193 records), together with random samples of the two remaining strata (200 of 784; 150 of 1604). Manual screening thus covered 543 records, 21.0% of those excluded, and recovered the two eligible studies added to the corpus.

A filter-bias check complemented the validation. Applying the empirical filter to the included studies retains 58 of 60 (97%), including all nine qualitative studies; the two studies it would have missed are quantitative.

### 3.5. Coding Scheme

Each study was coded on fourteen dimensions. Every dimension was anchored in a published taxonomy, which was either adopted unchanged, extended where the corpus required it, or derived from a theoretical architecture; Table 1 summarises the scheme and its sources. Two dimensions warrant comment. For D2a, coding practice forced one extension: unconfigured ChatGPT-class assistants and machine translation fit none of the four categories built from the 2007–2018 literature, so a fifth category was added. Classification followed the function the AI actually performed in each study, with named-tool defaults overridable by reported use. For D2b, multi-label coding was necessary because post-ChatGPT-era tools routinely combine several orientations in a single interface. Dimensions D9 to D14 locate each study within the language-learning domain and the instructional conditions of its intervention. For D10, a learning theory was counted only where it framed the study or motivated the intervention design; citing Flavell, Brown, Schraw or Zimmerman to define metacognition establishes a construct rather than a design rationale and was recorded but not counted, a rule that reduces the apparent theoretical coverage of the corpus by more than half. For D13, the coding question is not what the tool is called but who executes the meta-level operation, judged from the reported task sequence rather than from the authors’ characterisation of their tool.

### 3.6. Coding Procedure and Reliability

Coding covered the 71 full texts obtained at initial selection because eligibility was itself a coded judgement: the 13 full-text exclusions reported above could be made only after each article had been read and coded. The first and corresponding authors independently coded all 71 articles from the full texts using the written manual, with each author blind to the other’s assignments; the third author determined every disagreement, and the adjudicated codes constitute the analysed dataset. The first author began with a stratified random pilot of 14 articles (19.7% of 71), revised the decision rules and coding form, and then coded the remaining full texts. Following reviewer concerns about the reliability of the initially single-coded dataset, the corresponding author completed an independent second pass; all agreement statistics below compare these two passes. The two studies recovered through the recall validation in Section 3.4 were coded under the same procedure. Among dimensions added after the protocol, D9–D12 and the regulation sub-processes were coded by the first author alone. The regulatory-locus dimension (D13) and instructional conditions (D14) were independently recoded by the first and corresponding authors; final D14 values were settled by consensus, with the third author determining unresolved disagreements. On the pilot batch, agreement was substantial to almost perfect for most dimensions but lower for inclusion (κ = 0.58) because the boundary between metacognition proper and generic self-regulated learning was under-specified. The third author adjudicated the 16 pilot disagreements individually, and each decision was converted into a written rule. After the rules were fixed and the pilot batch recoded, agreement across all 71 double-coded articles was almost perfect: κ = 0.95 for inclusion, 0.97 for the role of metacognition, 1.00 and 0.96 for first- and second-order design, and 0.89 for direction of evidence. Reliability for the two dimensions developed in this review is reported separately: κ = 0.89 for AI application type, including the added fifth category, and κ = 0.87 for the multi-label functional-orientation scheme. For other multi-label dimensions, mean per-label κ and Jaccard agreement (in parentheses) were 0.82 (0.95) for metacognitive facet, 0.98 (0.99) for measurement modality, and 0.93 (0.99) for educational level. Treating entries flagged for adjudication as a separate category lowered these values by 0.02–0.04 without changing their interpretation. For the regulatory-locus dimension recoded under the provenance rule, the two authors agreed on 17 of 18 contrast-based studies (κ = 0.90; κ = 0.71 for the 26 studies recoded before reclassification and κ = 0.32 in the blind pilot under the original rule). Before the rule was tightened, independent coding of the instructional conditions yielded κ = 0.42 for explicit scaffolding, 0.80 for duration, and 0.42 for guidance. Subsequent strict recoding was completed by consensus, with the third author determining disagreements; no κ is claimed for those final values.

Study quality was appraised with the Mixed Methods Appraisal Tool (MMAT, 2018 version; Q. N. Hong et al., 2018), a validated instrument designed for reviews that include quantitative, qualitative, and mixed-methods studies together. The MMAT assigns each study to a design-specific branch—qualitative, randomised controlled, non-randomised, quantitative descriptive, or mixed methods—and applies five criteria appropriate to that tradition after two screening questions common to all studies. Each criterion is rated yes, no, or cannot tell, and the developers advise reporting the rating profile rather than collapsing it into a single score. The same double-appraisal procedure used for coding was applied, with design branches assigned from the coded designs (branch agreement between appraisers: 58/58 on the articles double-appraised before recall validation; the two recovered studies were appraised under the same rules, with disagreements determined by the third author). Overall agreement was 87.7% (κ = 0.70; the two screening items, with near-zero variance, are reported as raw agreement, 0.98 and 0.95, since κ degenerates under invariance). Disagreements were resolved by three adjudication rules fixed before the final ratings were consolidated—unreported quantitative–qualitative divergence and unreported nonresponse analysis were rated “cannot tell”; verifiable internal statistical contradictions were rated “no” on component quality—with remaining cases taking the more conservative rating. Ratings appear in Appendix A (Table A1).

### 3.7. Design-Based Evidence Strata and Synthesis

The strength of a directional inference depends on the study design. Because the corpus mixes randomised trials, single-group interventions, correlational surveys, and qualitative case studies, every directional code (D6) was stratified before synthesis into one of four design-based evidence strata. S1, contrast-based, denotes studies in which an AI-absent comparison exists, either between groups or within participants, against a no-AI or business-as-usual condition; comparisons between AI and an alternative active intervention designed to deliver equivalent support and comparisons between two AI-enhanced conditions do not qualify, since neither isolates the presence of AI. S2 denotes single-group pre–post or case designs, including experimental studies that report no comparison group. S3 denotes correlational or descriptive survey designs, and also carries experimental studies whose only contrast is between two AI-enhanced conditions, since such a design supports description of how AI configurations differ but not estimation of what the presence of AI contributes. S4 denotes qualitative description. Scale-development studies, which estimate no effect at all, were recorded separately. The ordering represents only the strength of inference each design supports about an AI-related directional difference; it does not rank the scientific quality or value of individual studies. Qualitative designs in particular may furnish especially strong evidence about how learners experience, monitor and regulate their interaction with AI, questions on which a contrast-based design is often mute, even though they cannot estimate a causal effect. The strata are therefore distinct in purpose from the MMAT appraisal reported in Section 4.6, which evaluates how well each study meets the standards of its own tradition. Primary directional conclusions rest on S1 evidence, with S2 to S4 reported as corroborating context.

No meta-analysis was attempted. Measures were heterogeneous, most studies relied exclusively on offline self-report, and—as the Results show—the facet on which effect-size synthesis would be most informative was not measured in any included study. The synthesis is therefore configurative: dimensions are cross-tabulated with one another and with the design-based evidence strata, and representative studies illustrate the resulting patterns.

## 4. Results

Section 4.1 introduces the corpus, and Section 4.1, Section 4.2 and Section 4.3 collectively address RQ1 by describing technological eras, learner populations, language domains, research designs, and approaches to conceptualising and measuring metacognition. Section 4.4 addresses RQ2 by synthesising evidence on the relationship between AI use and specific dimensions of metacognition. Section 4.5 addresses RQ3 by examining the learner-, task-, technology-, and instructional-level conditions associated with positive metacognitive outcomes. Section 4.6 then evaluates the methodological quality that bounds these interpretations.

### 4.1. Study Characteristics

**Figure 2:**
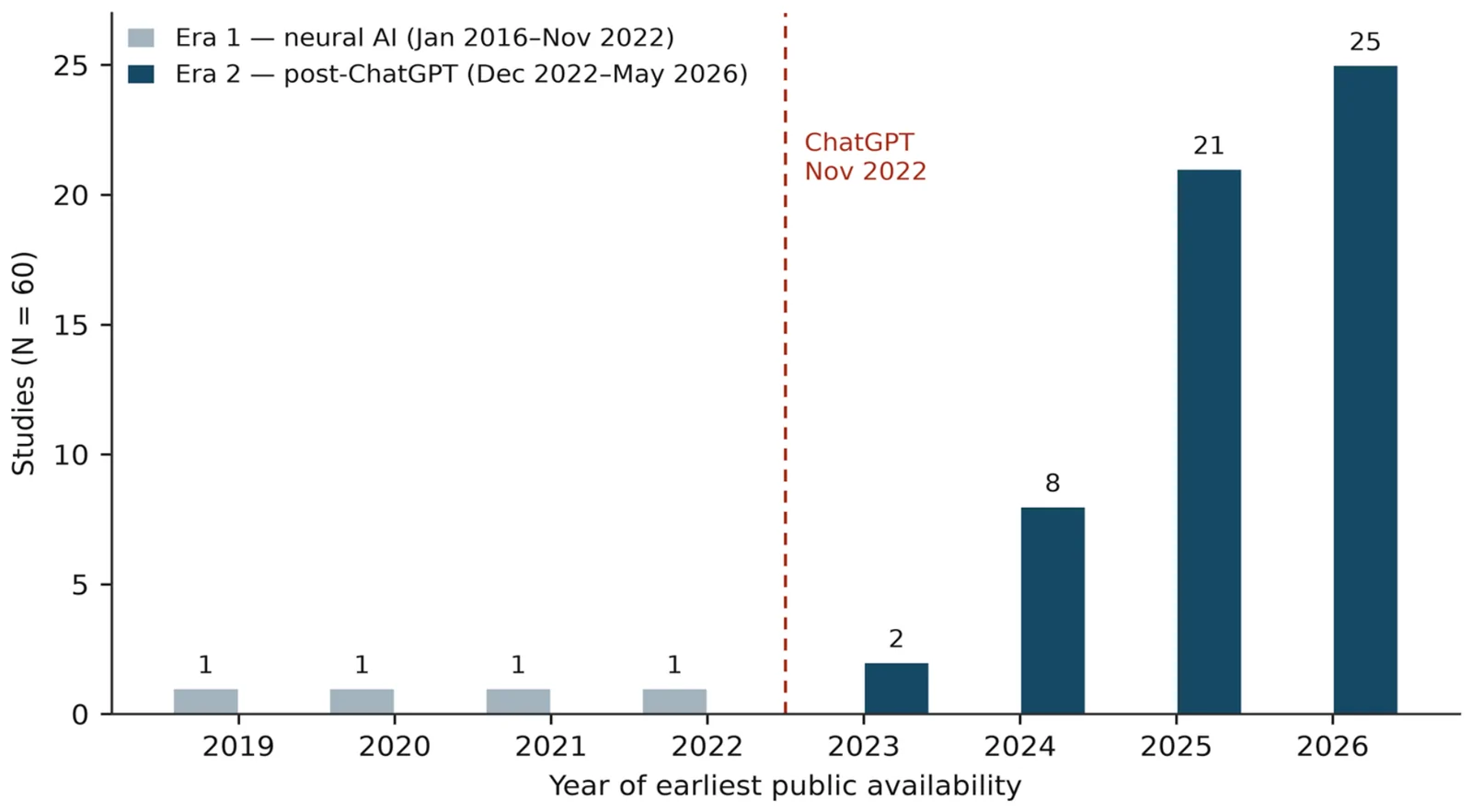
Included studies by year of earliest public availability (*N* = 60), by technological era. Both series belong to the single category “technological era” and therefore share one hue: Era 1 (neural AI, January 2016–November 2022) is shown at lower transparency and Era 2 (post-ChatGPT, December 2022–May 2026) at full transparency. The dashed line marks the public release of ChatGPT in November 2022, the boundary between the two eras.

Sixty studies met all criteria (Table A2), and their distribution over time is sharply asymmetric (Figure 2). Across the seven years of Era 1, only four eligible studies exist: two on mobile-assisted learning with an adaptive application, one on machine-translation post-editing, and one comparing feedback sources including automated feedback. All four were conducted in higher education and all measured metacognition by offline self-report; one of them (García Botero et al., 2021) also drew on platform usage logs. The remaining 56 studies fall in Era 2, with 25 published in the first five months of 2026 alone. Given this base, generational contrasts at coding depth are not statistically meaningful; the sparse neural-era record is itself informative, and the sections that follow characterise the post-ChatGPT corpus (*n* = 56) unless stated otherwise.

**Figure 3:**
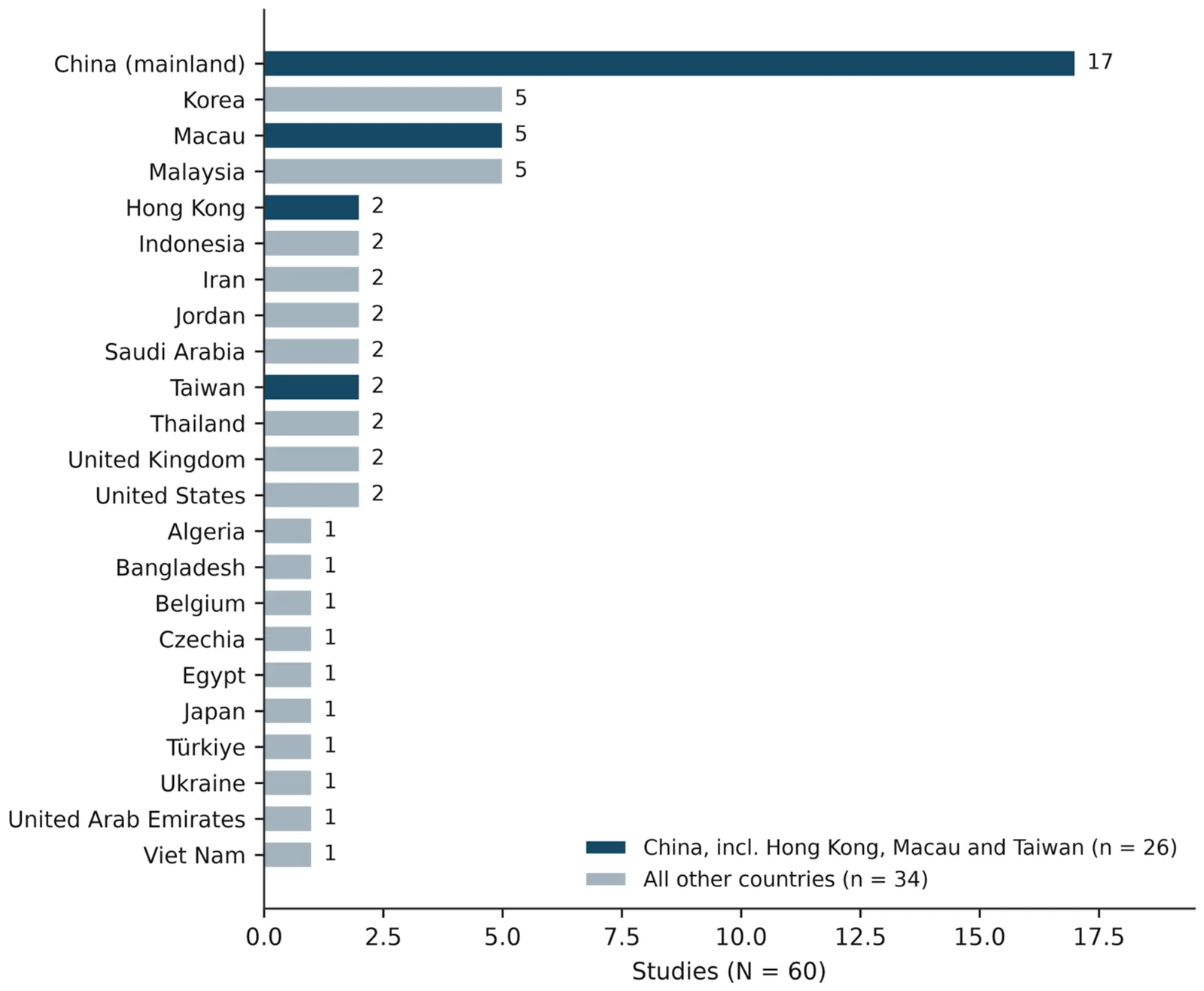
Included studies by country of the first author’s institution (*N* = 60). All bars belong to the single category “first-author country” and share one hue; the darker bars mark institutions in China, including Hong Kong, Macau and Taiwan (*n* = 26), and the lighter bars mark all other countries (*n* = 34).

The corpus is dispersed across publication venues and concentrated geographically. The 60 studies appeared in 44 journals, 32 of which contributed a single study, indicating limited consolidation around a shared set of publication venues. First authors were based in 23 countries, but 26 of the 60 studies (43%) were led from institutions in China, including Hong Kong, Macau and Taiwan, and only eight studies, from six countries, originated in Europe or North America (Figure 3). Section 5.8 reports an analysis separating the contribution of database coverage from that of field-level publication patterns to this concentration.

Under the classification of Gay et al. (2009), blended designs were the most common: 27 studies (45%) were blended, 24 (40%) quantitative, and 9 (15%) qualitative. Within the 51 studies carrying a quantitative component, the second-order distribution was experimental 25 (49%), correlational 13 (25%), survey 9 (18%), causal-comparative 2 (4%), and single-subject 2 (4%); the second-order scheme does not apply to the nine qualitative studies. Design and evidence stratum are closely but not perfectly aligned: 18 of the 25 experimental studies supplied the AI-absent comparison required for S1, while five were single-group or case designs carrying no comparison and two contrasted one AI condition against another; neither of the two causal-comparative studies met S1, and all correlational and survey studies fell to S3.

In terms of educational level, higher education dominates the sampled populations (55 of 60), with adult or vocational (6), senior secondary (2), and elementary (1) settings barely represented. This concentration limits the generalisability of the subsequent findings.

**Figure 4:**
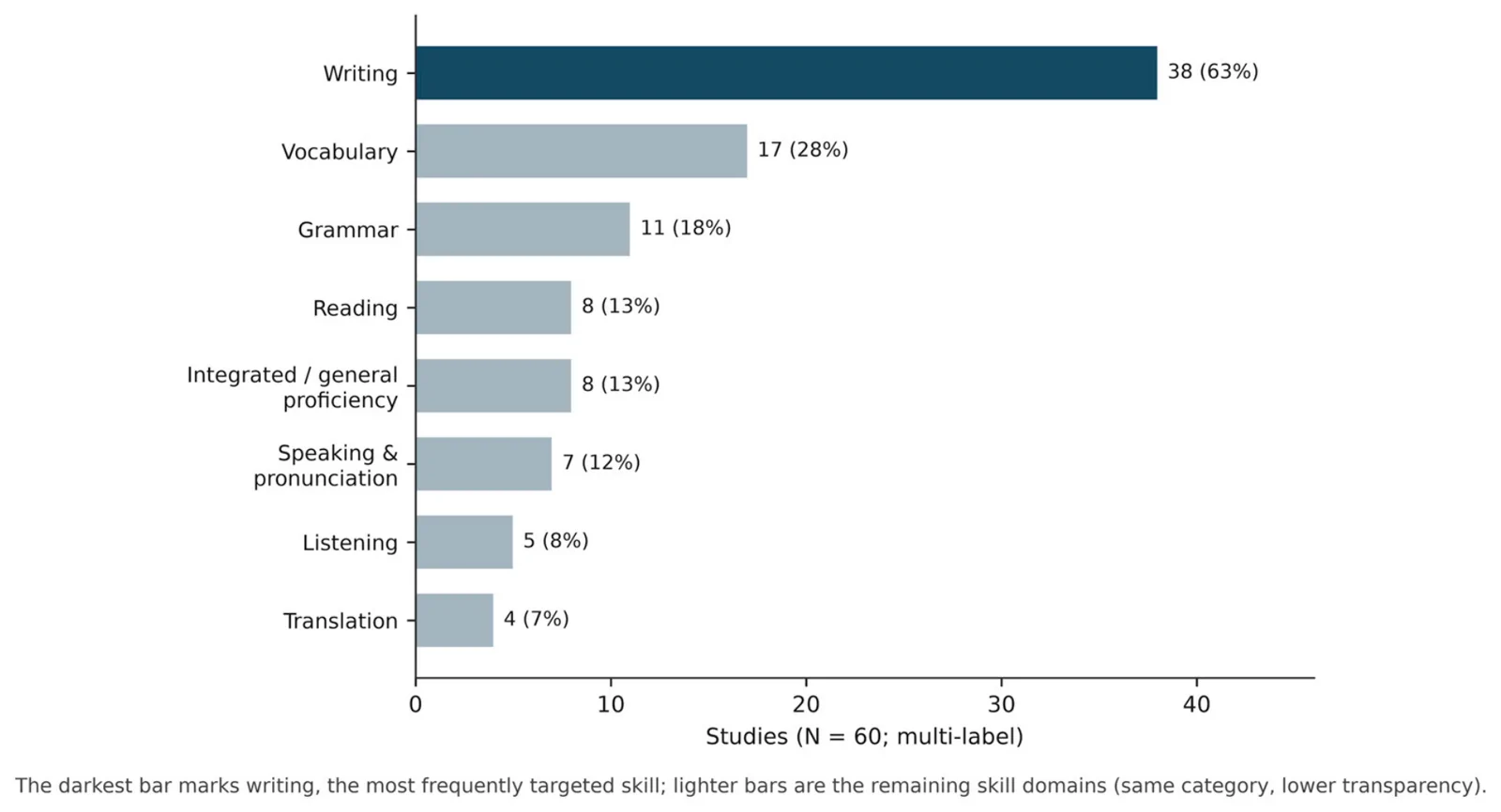
Language skill or content domain of the included studies (*N* = 60; multi-label). All bars belong to the single category “language skill or content domain” and share one hue; the darkest bar marks writing, the most frequently targeted skill (38 studies, 63%), and the lighter bars mark the remaining domains. Percentages are of the 60 included studies and, because the dimension is multi-label, exceed 100% in total.

**Figure 5:**
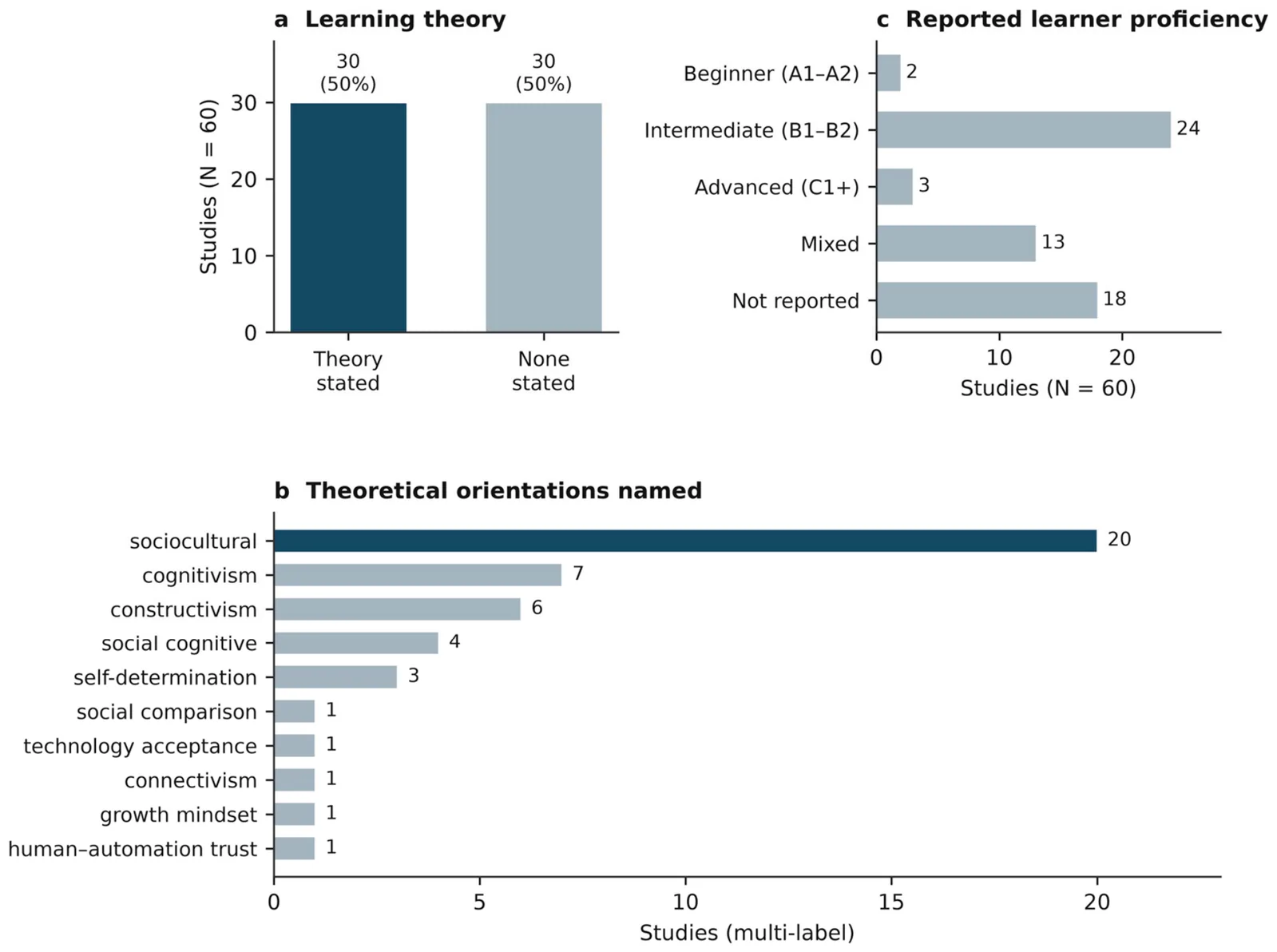
(**a**) Proportion of studies stating any learning theory; (**b**) theoretical orientations named (multi-label); (**c**) reported learner proficiency. In each panel the bars belong to a single category and share one hue; the darkest bar marks the most frequent level within that panel—theory stated in (**a**), sociocultural in (**b**), intermediate in (**c**)—and the lighter bars mark the remaining levels. In (**c**), “mixed” comprises studies reporting either a mixture of proficiency bands or a beginner-to-intermediate range.

The corpus is equally uneven across the language-learning dimensions that define the domain. Coded against the skill organisation of Golonka et al. (2014), writing was the target in 38 of the 60 studies (63%), followed by vocabulary (17, 28%) and grammar (11, 18%); reading (8), speaking and pronunciation (7), listening (5) and translation (4) together account for fewer studies than writing alone, and eight studies targeted general proficiency rather than a discrete skill (Figure 4). English was the sole target language in 54 studies; the remaining six comprised French, Spanish, Japanese and Chinese–English translation (one each), one bilingual English–Spanish activity, and one study in which learners selected the target language themselves. Learner proficiency, banded against the CEFR, was reported in 42 studies and left unreported in 18; where reported, it clustered at B1–B2, with a tail to A2 (Figure 5c).

Learning-theoretical commitments were coded against the three paradigms of Ertmer and Newby (1993), extended inductively with a sociocultural category and a residual category for other named theories. Half the corpus (30, 50%) framed its design and analysis without invoking any learning theory; sociocultural accounts predominated among the rest (20, 33%), followed by cognitivism (7), constructivism (6), social cognitive theory (4) and self-determination theory (3), with social comparison theory, technology acceptance, connectivism, growth mindset and human–automation trust named once each. No study drew on behaviourism. References to Flavell, Brown, Schraw, or Zimmerman that were used solely to define metacognition were not coded as evidence of a theoretical orientation, because such citations establish the construct rather than provide a rationale for the research design (Figure 5a,b).

### 4.2. What the AI Does: Application Types and Functional Orientations

Under the extended Zawacki-Richter taxonomy, assessment and evaluation applications led (27/56, 48%), followed by the added fifth category of content generation and general-purpose assistants (21, 38%). Intelligent tutoring (3), adaptive personalisation (4), and profiling and prediction (1) were marginal. The categories are best understood through the studies that typify them. M. F. Teng (2025b) illustrates the assessment-and-evaluation type: EFL undergraduates submitted drafts to ChatGPT for feedback, and the study examined how learners with differing metacognitive awareness evaluated and acted on that feedback—the AI evaluated learner-produced text, while the learner retained responsibility for composing it. Abdelhalim (2024) illustrates the fifth category: undergraduates used unconfigured ChatGPT throughout a research-methods course, from understanding research terminology and generating paper ideas to synthesising the literature and developing a proposal outline, so the system had no fixed instructional role and produced content on request. Aldamen et al. (2026) illustrates intelligent tutoring: ChatGPT provided strategy instruction within a structured reading lesson that was compared with a teacher-only condition using the same texts, strategy targets, and instructor. Geng and Yamada (2025) illustrate profiling and prediction, with a learning-analytics dashboard presenting learners’ own process data in an augmented-reality environment.

Functionally, diagnostic orientation appeared in 42 studies (75%), generative orientation in 25 (45%), and dialogic orientation in 14 (25%). These orientations cut across application types. J. Zhang and Zhang (2024) is purely diagnostic: automated writing evaluation returned trait-level judgements on learner essays without producing text. Hsu et al. (2024) is generative in a non-writing task, where an image-recognition system supplied the target vocabulary that learners were to retrieve. Dillon (2024) is dialogic, training learners to construct and test prompts and to sustain multi-turn exchanges in which ChatGPT also questioned the learner. Critically, 23 studies (41%) deployed tools carrying two or more orientations at once, and in 15 of them (27%) the combination was diagnostic together with generative; M. F. Teng (2025b) is representative, since the same ChatGPT session that evaluated a draft could also rewrite it. In the neural era, these functions generally appeared in separate tools; in the post-ChatGPT era, they can coexist within a single conversational interface. A learner can therefore move from requesting feedback to delegating the task through a subsequent prompt.

### 4.3. What Gets Measured: Facets and Modalities

**Figure 6:**
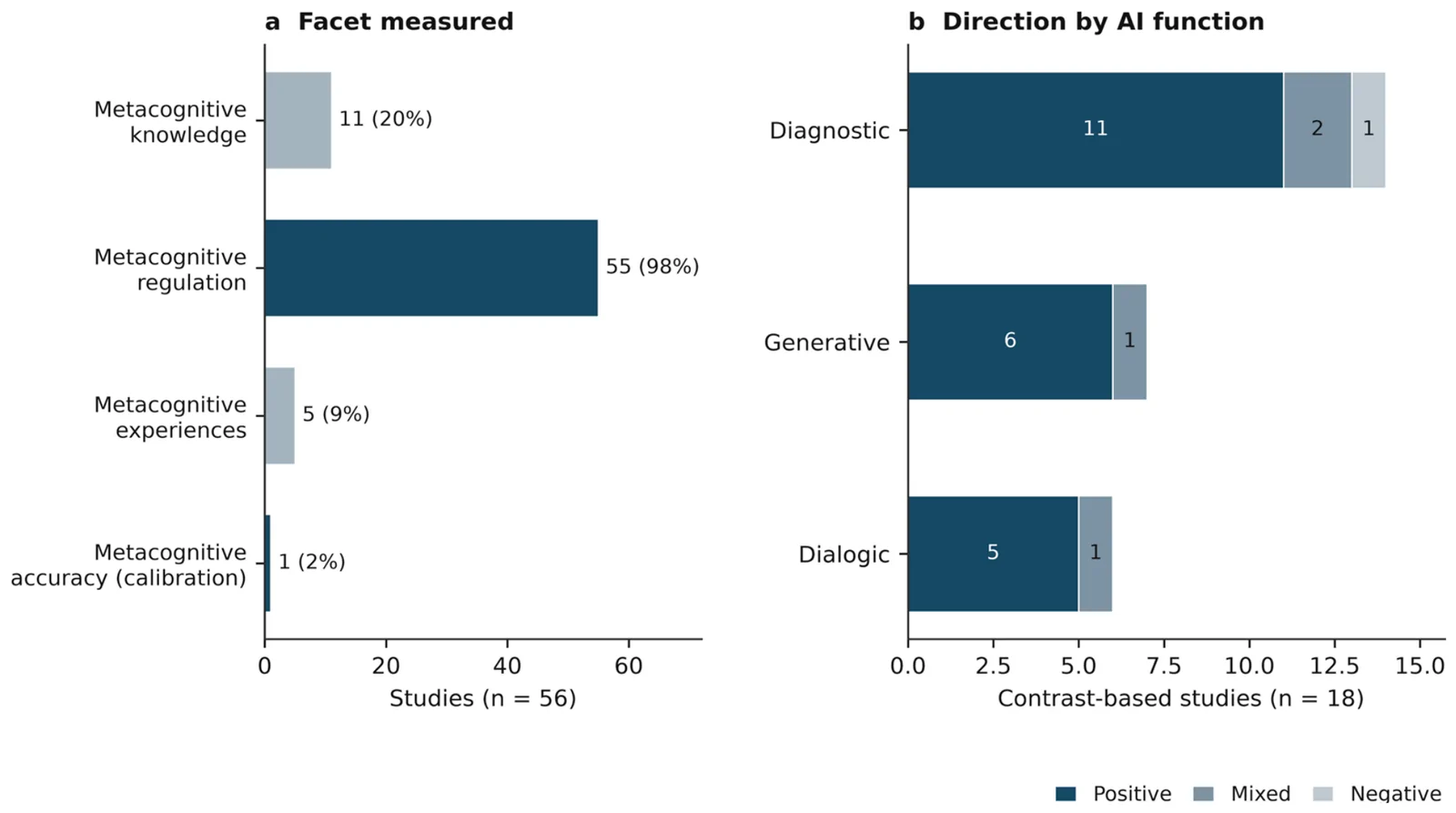
(**a**) Metacognitive facets measured in post-ChatGPT studies (*n* = 56); (**b**) direction of reported evidence by AI functional orientation, contrast-based studies only (*n* = 18). In (**a**) the bars belong to the single category “metacognitive facet” and share one hue: the darkest bars mark metacognitive regulation, the most frequently measured facet (55 studies, 98%), and metacognitive accuracy, measured in a single study (2%). In (**b**) the bars belong to the single category “direction of reported evidence” and share one hue, separated by transparency—most opaque for positive, intermediate for mixed, least for negative, as shown in the legend. Because functional orientation is multi-label, the three rows are not mutually exclusive and do not sum to 18.

Figure 6 shows that measurement is concentrated on a single facet. Metacognitive regulation was measured in 55 of the 56 post-ChatGPT studies (98%); metacognitive knowledge in 11 (20%); and metacognitive experiences in 5 (9%). Only one post-ChatGPT study measured metacognitive accuracy—including calibration, judgements of learning, overconfidence, or the illusion of competence (2%; Figure 6a)—and it was the sole such study in the full corpus of 60. This is the corpus’s most consequential measurement gap. Whether generative AI inflates learners’ assessments of their own ability is precisely the question that calibration paradigms exist to answer, and one study in sixty addresses a version of it, with a diagnostic feedback system rather than a generative one. That study (Appendix B: C40) paired item-level judgements of learning with performance on the same items and reported bias, root-mean-square error and a Goodman–Kruskal gamma coefficient alongside a calibration curve. It is a workable template rather than an exotic design—though a diagnostic one, so the generative-AI version of the question remains untested—and it shows that the instrumentation this literature lacks is neither costly nor unfamiliar. Elsewhere, the gap is not for want of raw material: in another included study (Appendix B: D22) a learner spontaneously compared his own estimate of his speaking level with the band the system assigned—the two ingredients of a calibration index—yet no accuracy statistic was computed there.

**Figure 7:**
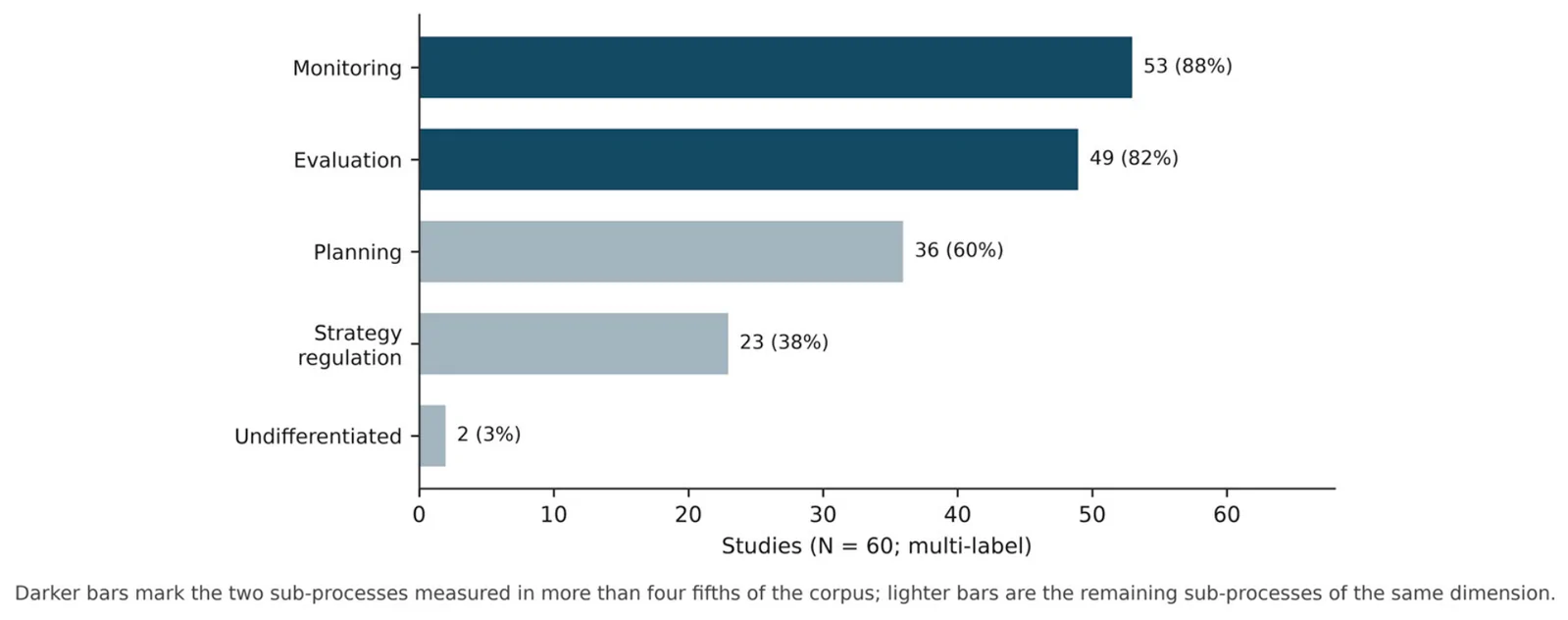
Metacognitive regulation sub-processes measured in the included studies (*N* = 60; multi-label). All bars belong to the single category “regulation sub-process” and share one hue; the darker bars mark monitoring and evaluation, the two sub-processes measured in more than four fifths of the corpus (53 studies, 88%; 49 studies, 82%), and the lighter bars mark the remaining sub-processes. Percentages are of the 60 included studies and, because the dimension is multi-label, exceed 100% in total.

Within metacognitive regulation, the sub-processes were unevenly represented. Coded against Brown (1987) and the regulation subscales of Schraw and Dennison (1994), monitoring appeared in 53 of the 60 studies (88%) and evaluation in 49 (82%), but planning in only 36 (60%) and strategy regulation—the adjustment, information management and debugging that follow a monitoring judgement—in 23 (38%); two studies reported an undifferentiated composite. The literature therefore measures problem detection far more often than the strategy regulation required to address the problem, even though generative systems can now supply that response on request. Modality compounds the problem: every post-ChatGPT study used offline self-report (56/56), only 19 (34%) triangulated it with online process data, and 37 (66%) relied on self-report alone (Figure 7).

### 4.4. Roles and Directions of Evidence

Metacognition functioned in study designs predominantly as an outcome of AI use (36/56, 64%) rather than as a strategic process enacted during AI use (20, 36%). The third position identified by Z. Zhang et al. (2025), metacognition as the framework guiding instructional design, was occupied by no post-ChatGPT study at all. Across the full corpus of 60, it appears exactly once, in a pre-generative study that trained learners in self-regulation and scaffolded their use of a mobile application (Appendix B: A14); that study measured participation and language test scores rather than metacognition itself, and is carried in Table 2 without a direction code. The post-ChatGPT literature has therefore not tested interventions designed explicitly around a metacognitive model. Reported directions skew positive, but the skew is carried disproportionately by weaker designs. Stratifying by design-based evidence stratum (Table 2), the 16 post-ChatGPT contrast-based studies (S1) reported 13 positive, 2 mixed, and 1 negative result, whereas correlational and descriptive designs added 11 positive with almost no counter-evidence and qualitative reports a further 9 positive.

Representative studies clarify these directional categories. Among positive S1 findings, Aldamen et al. (2026) compared a teacher-mediated, AI-assisted strategy lesson with a teacher-only lesson and a business-as-usual section, finding the largest gain in metacognitive awareness in the AI-assisted condition. Du (2025) likewise reported improved goal setting, monitoring, and reflection after a semester-long conversational-agent intervention. The negative S1 finding and the negative association outside S1 differ in both design and mechanism. Dhanapal et al. (2026) traced growing dependence on automated writing assistants across a 16-week randomised contrast, whereas Tekir (2026), using a self-selected design without an AI-absent comparison, found that heavier generative-AI use was associated with cognitive offloading and weaker metacognitive engagement in Turkish EFL writing. Mixed findings generally reflected divergence among sub-indicators: Lai et al. (2026), for example, found gains in some components of metacognitive knowledge but no difference in problem identification across task-offloading configurations. Null findings were rare. S. Wang et al. (2026) found that self-efficacy in using large language models did not significantly predict metacognition (β = 0.06, *p* = 0.11), although metacognition predicted subsequent learning behaviour. Four additional studies, including scale-development or validation studies such as Yao and Fan (2026) and M. F. Teng (2026), estimated no effect.

Within S1 evidence, counted at the level of functional orientations, direction did not differ detectably by function: diagnostic 11 positive/2 mixed/1 negative, generative 6/1/0, dialogic 5/1/0 (Figure 6b). This null contrast should not be read as evidence that generative substitution is metacognitively benign, because the corpus is poorly configured to detect the harm in question. Detecting inflated self-assessment would require, at minimum, an AI-absent comparison, a process-level record of what the learner actually did, and a measure of judgement accuracy. Eighteen studies provide the first, 20 provide the second, six provide both (five post-ChatGPT), and exactly one, Hu and Yordchim (2025), provides the third. Only one study combines the measurement features needed to identify this form of metacognitive harm; the remaining fifty-nine do not. For all but one study, the corpus records what learners say about their monitoring without establishing whether that monitoring is accurate.

### 4.5. Conditions Associated with Positive Metacognitive Outcomes

**Figure 8:**
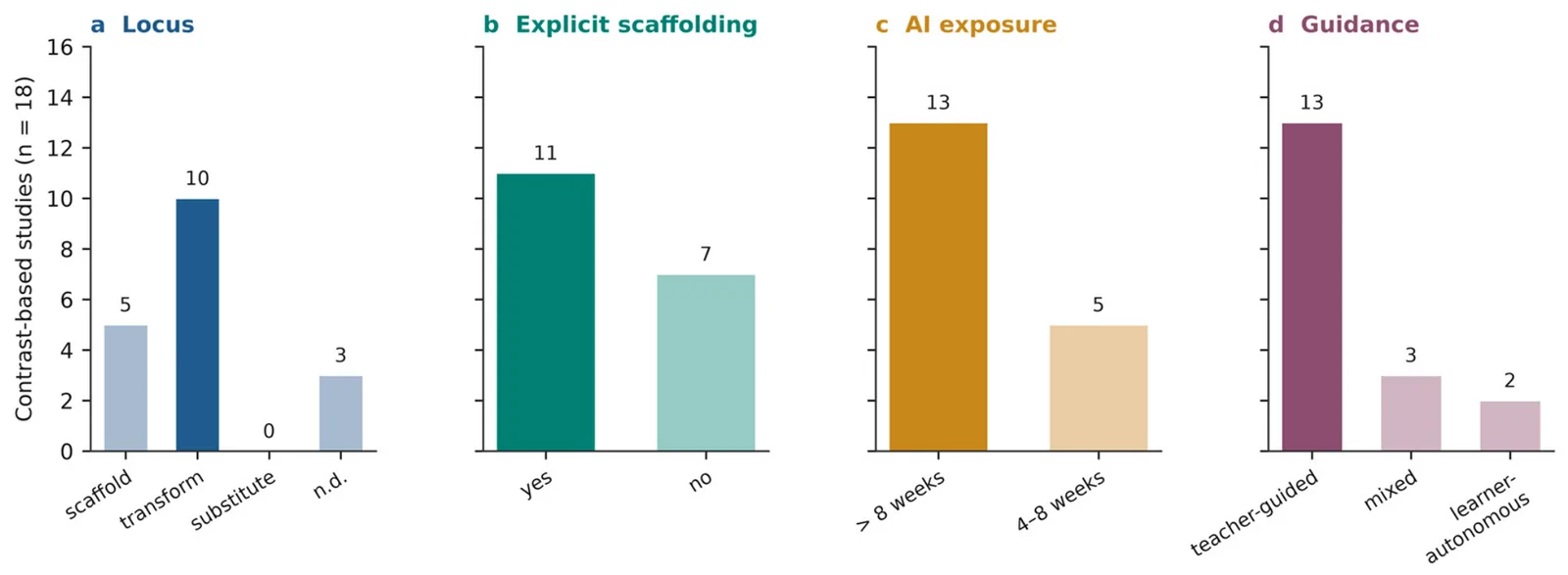
Instructional conditions under which AI was used (contrast-based studies, *n* = 18). n.d. = locus not determinable. The four panels represent four different instructional dimensions rather than four levels of one, so each panel is given its own hue: locus (**a**), explicit metacognitive scaffolding (**b**), AI-exposure duration (**c**) and guidance (**d**). Within each panel the bars belong to that single dimension and are separated by transparency, the darker bar marking the more frequent level. The zero-height bar in (**a**) records that no contrast-based study deployed AI as a substitute, and is retained so that the empty cell remains visible.

Examining the conditions associated with positive metacognitive outcomes requires variables that can be identified directly from the study reports. Two were coded for this purpose. The first, following the meta-level–object-level architecture of Nelson and Narens (1990), records which party performs the regulatory operation: the AI may prompt the learner to plan, monitor or evaluate while leaving the operation to be executed by the learner (scaffold); it may take over part of the operation while the learner retains judgement over the result (transform); or it may execute the operation and deliver its product (substitute). The second records the instructional conditions surrounding use—whether explicit metacognitive scaffolding was embedded in the intervention, how long the AI-exposure period ran, and whether use was teacher-guided or learner-directed. Both dimensions were coded under the evidence-provenance rule described in Section 3.6, and both are reported for the 18 contrast-based studies, the set in which they enter the analysis; they are not reported for the remaining studies, because the audit described below showed the original procedure unreliable and the dimensions carry no analytic role outside the contrast-based set. Among the 18, five deployed AI as a scaffold and ten as a transformation of the regulatory division of labour; three designs do not specify the division, and none deploys AI as a substitute. Explicit metacognitive scaffolding was embedded in eleven of the 18, use was teacher-guided in thirteen, and the AI-exposure period exceeded eight weeks in thirteen (Figure 8).

**Figure 9:**
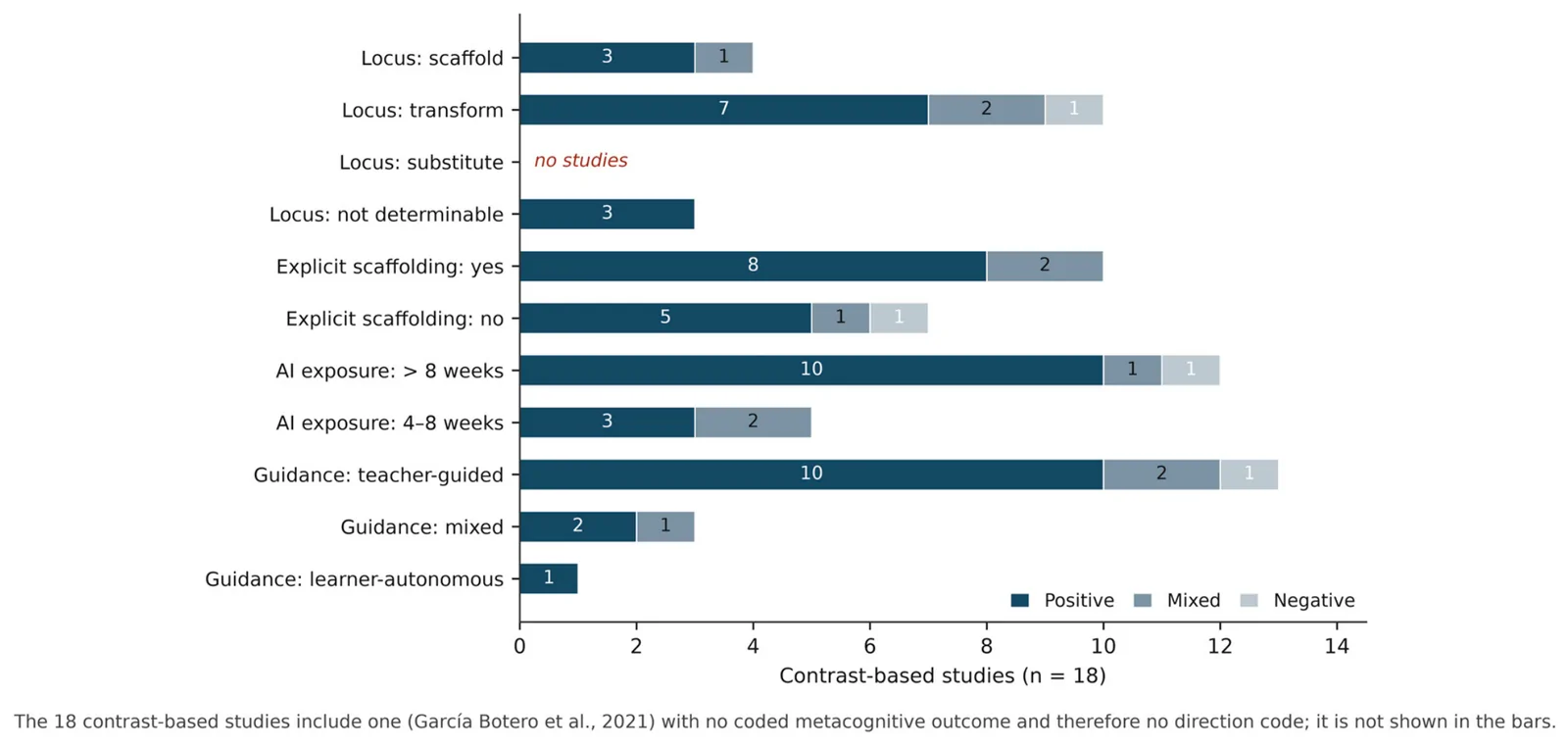
Direction of reported metacognitive evidence within each instructional condition (contrast-based studies, *n* = 18). The figure displays the counts cross-tabulated in Table 3. All bars belong to the single category “direction of reported evidence” and share one hue, separated by transparency—most opaque for positive, intermediate for mixed, least for negative, as shown in the legend. Within each block the rows total the number of contrast-based studies carrying that condition. “Locus: substitute” is empty because no contrast-based study deployed AI as a substitute, and no contrast-based study reported a null direction, so that category does not appear. One of the 18 studies (García Botero et al., 2021) measured no metacognitive outcome and therefore carries no direction code; it contributes to the condition totals but appears in none of the bars.

The conditions were cross-tabulated with evidence direction across the 18 contrast-based studies, including two pre-generative cases (Table 3; Figure 9). One of the 18, García Botero et al. (2021), measured no metacognitive outcome and therefore contributes to the condition totals but has no direction code. The analysis is descriptive because the coding history precludes a configurational solution. The locus dimension was formulated after the protocol, and a provenance audit conducted in response to review found that some initial assignments relied on Results or Discussion text: two of fourteen studies coded as scaffold, three of ten coded as transformation, and both studies coded as substitution. The two cases supporting the previously reported pattern had therefore been classified according to their findings rather than their deployment of AI. Coding direction before formulating the locus dimension prevented one form of contamination but not this reverse pathway. We consequently introduced an explicit provenance rule that admitted only Method, intervention, procedure, and system-description text. The first and corresponding authors independently recoded the 26 studies then classified as contrast-based, increasing agreement from κ = 0.32 in the blind pilot to κ = 0.71. Of these 26 studies, full-text re-inspection removed eight because none included an AI-absent comparison: two compared AI users with one another (Tekir, 2026; Abdelhalim, 2024), two compared different AI-enhanced conditions, and four reported no comparison group. Among the 18 retained studies, the authors agreed on 17 classifications (κ = 0.90), and neither author classified any study as substitution. The increase from κ = 0.71 primarily reflects reclassification rather than the provenance rule because three of the four earlier disagreements occurred in studies subsequently removed from the stratum. The third author determined the remaining disagreement at the scaffold–transformation boundary. Table 3 reports the first author’s values; the corresponding author’s alternative distribution (4 scaffold, 11 transformation, 3 not determinable) appears in Supplementary File S5. Of the two studies initially classified as substitution, one falls outside the contrast-based stratum and the other is classified as transformation because the tool flags problems and proposes revisions while the learner decides whether to adopt them.

No contrast-based study in the corpus assigns the learner’s regulatory operation to AI, which limits the comparison reported in Table 3. Evidence direction does not vary meaningfully by locus: three of four scaffold deployments and seven of ten transformations report positive results, compared with 76% (13 of 17) across the contrast-based studies with a direction code. The other coded conditions show similarly limited differentiation. Explicit metacognitive scaffolding accompanies a positive result in eight of ten studies with direction codes, compared with five of seven without it. Ten of twelve interventions lasting more than eight weeks report positive results, compared with three of five lasting four to eight weeks; teacher-guided use yields ten positive results among thirteen studies. Target skill also fails to distinguish outcomes: writing, the most frequently studied domain, produces a non-positive result in three of its ten contrast-based studies with a direction code, close to the proportion for the full set. No coded condition clearly separates positive from non-positive findings, and a single negative case cannot support analysis of the conditions under which metacognition deteriorates. The evidence is therefore least developed for the condition of greatest practical importance: no contrast-based design tests what happens when the system performs the regulatory operation instead of prompting the learner to perform it. Lai et al. (2026) comes closest by assigning three intact classes to conditions that differed in whether the learner or AI drafted and evaluated; it is the only study in the corpus to examine this division directly.

The locus dimension was formulated after the protocol and recoded under the evidence-provenance rule described in Section 3.6. Agreement between the two authors was κ = 0.71 for the 26 recoded studies and κ = 0.90 for the 18 retained after full-text re-inspection of design. The single residual disagreement, concerning the scaffold–transformation boundary, is documented in Supplementary File S5 and was determined by the third author; the table reports the first author’s values. Eight studies were removed from the contrast-based stratum because they lacked an AI-absent comparison. García Botero et al. (2021) contributes to the condition totals but has no direction code. Necessity and sufficiency parameters are not reported because the analysis is descriptive, the 18 cases leave most logically possible combinations unobserved, and the substitution category is empty. Du (2025) reports an AI-exposure period of 16 weeks in the Method and 8 weeks in the Limitations; the Method value was coded.

### 4.6. Methodological Quality

Following the guidance of the instrument’s developers, the appraisal is reported at criterion level in Table A1 and is not collapsed into an overall score. Three patterns are systemic rather than idiosyncratic. The first concerns sampling in the quantitative descriptive branch. Of its fourteen studies, not one was judged to have sampled a population it could represent: eight were rated no and six cannot tell. Nonresponse compounds this. Only three permitted a judgement that the risk of nonresponse bias was low, and in eleven, the question could not be answered at all, because valid-response counts were reported while comparisons between responders and non-responders were not. The second concerns integration in the twenty-seven mixed-methods studies. Its presence is not the difficulty: a rationale for the design was given in twenty-two and the components were judged effectively integrated in twenty-three. Divergence between the quantitative and qualitative strands is another matter. It was addressed adequately in eleven, and in sixteen, no appraisal could be made, because these studies report convergence only and leave no way to distinguish an absence of divergence from an absence of examination. The third pattern is the most consequential. In sixteen of the twenty-seven mixed-methods studies, the components were judged not to meet the quality standards of their own traditions, and nineteen studies across the corpus failed the final criterion of their respective branch. A recurring reason for that failure is the combination of fluent methodological language with internally inconsistent statistics: impossible degrees of freedom, irreconcilable sample sizes, and analyses announced in the Method that never appear in the Results. Because this judgement is recorded in Table A1 rather than held separately, the sensitivity check can be anchored to the published grid. Five of the nineteen studies failing the final criterion are contrast-based. Excluding all five leaves 13 contrast-based studies, of which 10 report positive and 2 mixed directions and one carries no direction code, a positive share of 83% among the 12 with a directional result against 76% for the full contrast-based set, so no directional conclusion rests on them. Eight studies met all seven criteria, and these include the corpus’s only fully process-triangulated designs. These studies show that the relevant methodological standards are attainable; the corpus-level gaps reflect prevailing practice rather than infeasibility.

## 5. Discussion

This review examined how empirical research published between 2016 and May 2026 conceptualised and measured metacognition in AI-supported second and foreign language learning. Rather than estimating an average effect or deciding whether AI is beneficial or harmful, it assessed which metacognitive constructs the field has measured, which modalities and designs it has used, and what inferences those designs support. This distinction is necessary because metacognition comprises several related constructs and AI mediation encompasses different interventions. A system that evaluates learner-produced text engages regulatory processes differently from one that produces the text for the learner. Interpreting the evidence therefore requires identifying which combinations of construct, technology, and division of regulatory responsibility have actually been studied.

The corpus itself carries an initial finding. Sixty eligible studies were published between 2016 and May 2026, but only four appeared in the pre-ChatGPT portion of the review window, against 56 in the three and a half years after the model’s public release, with 25 in the first five months of 2026 alone. The literature is thus overwhelmingly recent, geographically concentrated, and produced under conditions of rapid publication. The sections below interpret this pattern and the coded findings in the order of the research questions.

### 5.1. Concentration of the Evidence Base in a Single Era

The concentration of eligible research in the post-ChatGPT era is not explained by the availability of relevant technology. Throughout the neural era, automated writing evaluation, speech scoring, adaptive review systems, and machine translation were all in routine use in language classrooms, and all of them intervene in processes that metacognition ordinarily coordinates. Automated writing evaluation, in particular, externalises evaluation—the function that metacognitive regulation performs internally—and would have been a natural site for studying whether such externalisation supports or supplants learners’ own monitoring. Yet only four studies in seven years measured learner metacognition under any form of AI mediation.

Research attention may have followed perceived risk more closely than technological availability. Earlier diagnostic tools evaluated learner-produced work, but learners still wrote the essay, planned revisions, and decided which feedback to accept. Generative systems can perform each of these activities, allowing learners to obtain a competent product without necessarily engaging the processes that the task is intended to develop. This possibility may help explain the growth of metacognition-focused L2 research after 2023 and aligns with experimental concerns about metacognitive laziness (Y. Fan et al., 2025). It also warrants caution when interpreting the evidence: a literature that emerged partly in response to perceived risk may frame its questions and outcomes accordingly.

### 5.2. The Limits of Inherited AI Taxonomies

Coding AI application types required extending the four-category taxonomy of Zawacki-Richter et al. (2019). Twenty-one of the 56 post-ChatGPT studies used unconfigured ChatGPT-class assistants or machine translation, neither of which fit categories derived from the 2007–2018 literature; a fifth category was therefore added. This mismatch reflects a substantive change in how AI functions. Taxonomies developed for single-function systems assume stable instructional roles, such as tutoring or assessment. General-purpose generative tools have no fixed role because the learner’s prompt determines their function. The inherited taxonomy therefore cannot describe whether the system provides a judgement for the learner to act on or acts on that judgement itself; the same assessment-and-evaluation tool may do either across successive requests. Coding who performs the meta-level operation provides a more informative account of study design. However, the corpus offers no variation on the most consequential category: every contrast-based study leaves the regulatory operation with the learner, so the evidence base has not tested the distinction that the inherited taxonomy fails to capture.

The same instability appeared at the level of functional orientation, where 41% of post-ChatGPT studies used tools carrying two or more orientations at once and 27% combined diagnostic with generative functions in a single interface. Nguyen et al. (2026), comparing earlier and generative AI across educational domains, found earlier systems offering analytics-driven adaptation and generative systems offering dialogic scaffolding. The L2-specific evidence here suggests a further point: these are not merely different affordances distributed across different tools, but co-present affordances within one interface. A learner who submits a draft for evaluation and a learner who requests that the draft be written interact with the same system through the same channel, and the boundary between the two is set by the learner’s own regulatory decision. This has a direct implication for how AI use should be operationalised in future research, which we take up below.

### 5.3. The Absence of Calibration Measures

The review’s most consequential result is the absence of calibration measures. Although 98% of the 56 post-ChatGPT studies measured metacognitive regulation, generally through offline self-report, only one study in the full corpus measured metacognitive accuracy. The literature thus concentrates on the most readily elicited facet while largely omitting the facet most directly implicated by generative AI.

Metacognitive accuracy or calibration concerns whether learners’ judgements about their own learning correspond to their actual performance (Nelson & Narens, 1990). It is measured through well-established procedures—judgements of learning compared against subsequent performance, postdiction accuracy, and indices of bias and resolution—that have been in use for decades. The question these procedures answer is precisely the question that generative AI raises: when a system can produce competent output on request, do learners’ assessments of their own competence remain anchored to what they can do unaided? No study in this corpus provides evidence on this point. The gap is not attributable to infeasibility: in one included study (Q. Xu & Wang, 2025) a learner spontaneously compared his own estimate of his speaking level with the band the system assigned—the two components of a calibration index—yet no accuracy statistic was computed there.

Measurement modality compounds the problem. All 56 post-ChatGPT studies used offline self-report, 19 (34%) triangulated it with online process data, and 37 (66%) relied on self-report alone. Veenman et al. (2006) report that offline and online metacognitive indicators correlate only weakly, and the divergence matters here for a specific reason: if generative AI distorts learners’ perception of their own cognitive engagement, self-reported gains in planning and monitoring are precisely the outcome such distortion would produce. The L2 field’s well-developed self-report instruments (Vandergrift et al., 2006; Mokhtari & Reichard, 2002) have supported a productive research tradition, but their availability appears to have channelled research toward the facet they measure. That channelling is specific to the AI literature rather than to L2 research at large: Z. Li et al. (2026) were able to synthesise seven studies of L2 student writers’ metacognitive experiences, a facet represented in only five studies in the present corpus.

### 5.4. The Positive Skew in Reported Findings

Taken at face value, the corpus appears reassuring: positive findings outnumber negative ones by a wide margin. Stratification by design-based evidence stratum qualifies that impression. More than half of the positive directional evidence comes from correlational, descriptive, or qualitative designs in which no AI-absent comparison was available. Among the 16 post-ChatGPT contrast-based studies the balance is 13 positive, 2 mixed, and 1 negative, and among the five post-ChatGPT studies that combine a contrast design with process-level data the positive margin narrows further.

Two additional considerations qualify the apparent positive skew. First, evidence direction did not differ detectably by functional orientation within the contrast-based studies, but the comparison has low power. More importantly, only one study measured the outcome through which generative substitution would be expected to operate, and it examined a diagnostic rather than generative system. Second, the negative studies were methodologically substantive. Dhanapal et al. (2026) used a randomised contrast, and Yan and Zhang (2024) documented failures of metacognitive regulation using keystroke and screen-recording data coded with an established scheme. Tekir (2026), although based on self-selected use without an AI-absent comparison, also reported a negative association. The corpus therefore leaves the question unresolved rather than establishing a positive effect. Until calibration measures are incorporated into these designs, an absence of observed harm cannot support the conclusion that harm is absent.

### 5.5. Metacognition as Label Rather than Construct

A subsidiary but consequential observation emerged from full-text screening: in seven of the thirteen articles excluded at that stage, metacognition appeared only in titles, abstracts, or keywords with no measurement, and in two more, a generic self-regulated-learning composite carried no metacognitive conceptualisation; several further included studies contained internally inconsistent statistics that our page-anchored coding flagged. This pattern is systemic rather than attributable to individual studies: as metacognition gains prominence in generative-AI research, it is increasingly used as a framing term without corresponding construct-level operationalisation, reproducing the conflation identified by Dinsmore et al. (2008). Reviews based only on titles and abstracts may therefore include studies that name but do not assess the construct. Full-text screening against explicit operational criteria is consequently necessary.

### 5.6. Selective Inheritance from L2 Metacognition Research

Compared with the L2 metacognition literature reviewed in Section 2, the present corpus retains some research priorities while leaving others largely unexplored. Earlier research distributed attention across listening, reading, writing, and vocabulary, and its most developed instrument—the Metacognitive Awareness Listening Questionnaire, validated with 966 learners by Vandergrift et al. (2006) and elaborated by Vandergrift and Goh (2012)—focuses on listening. Under AI mediation, this distribution reverses: writing accounts for 38 of 60 studies, whereas listening accounts for five (Figure 4). This pattern is unlikely to reflect a judgement that metacognition matters more for writing. It may instead follow the output most readily produced by generative systems. Writing represents 63% of the present corpus but 34% (93 of 272 studies) in Y. Wang et al.’s (2026) twenty-year mapping of AI in language learning. This interpretation remains provisional because the review coded what researchers studied, not why they selected those domains. Nevertheless, the concentration on text-based production has left two decades of listening-strategy instrumentation largely unused at a time when AI can increasingly mediate the comprehension processes that learners are expected to monitor.

A second inheritance was declined rather than selected. Raoofi et al. (2014), synthesising 33 studies from the pre-AI period, concluded that metacognition contributes to L2 attainment but that the contribution varies substantially with learner and contextual characteristics; identifying those moderators was the agenda they set. The present corpus cannot take it up. Learner proficiency, the most obvious of those moderators, is unreported in 18 of the 60 studies, and where reported it clusters so tightly at B1–B2 (Figure 5c) that variation is unavailable even in principle. Cai (2024) showed with 2126 secondary-school learners across three annual waves that metacognitive strategy use is not a fixed attribute but shifts over time; yet 19 studies here do not report how long their intervention ran at all, and among the contrast-based studies that do, 10 of 12 longer than eight weeks and 3 of 5 shorter reported a positive result—a difference the available cases cannot resolve either way. A literature that inherited a developmental question has produced a predominantly cross-sectional answer.

What the corpus did inherit, and confirms, is the value of making regulation explicit. F. Teng (2016) found that embedded metacognitive instruction improved Chinese EFL learners’ writing and regulation of cognition, with effects persisting beyond the instructional period. A decade later, evidence from a different technological context shows a similar pattern: among studies with explicit metacognitive scaffolding, eight reported positive findings, two mixed findings, and one had no direction code, compared with five positive, one mixed, and one negative finding among studies without explicit scaffolding (Table 3). This continuity is consistent with the possibility that pedagogy, rather than the technology itself, is the active ingredient. The comparison cannot establish that conclusion because embedded scaffolding, teacher guidance, and methodological quality co-occur in this corpus and cannot be separated at *n* = 18.

The corpus also inherits a degree of theoretical thinness that is less visible in the review literature. Several syntheses discussed in Section 2.6 are explicitly theory-anchored: Chang and Sun (2024) use Zimmerman’s cyclical model, Nguyen et al. (2026) use the COPES architecture, and Z. Zhang et al. (2025) use a tripartite account of SRL. Yet half of the primary studies reviewed here state no learning theory (Figure 5a); none invokes behaviourism, and only seven invoke cognitivism. The reviews are therefore more explicitly theorised than the underlying primary literature. This distinction limits interpretation: when a study neither adopts a theory of learning nor measures metacognitive accuracy, a positive self-report supports the conclusion that learners perceived themselves as more strategic, but not that a specific mechanism was engaged.

Finally, one inheritance runs in the opposite direction. Section 2.1 treats metacognitive regulation as a cycle in which monitoring leads to repair. The corpus measures the first half of that cycle far more often than the second: monitoring in 88% of studies and evaluation in 82%, but strategy regulation—the adjustment, information management and debugging that a monitoring judgement is supposed to trigger—in only 38% (Figure 7). This is the sub-process that a generative system is most plausibly implicated in, and it is the one the field is least equipped to observe. Z. Li et al. (2026) report a similar lack of attention to metacognitive experiences outside the AI literature, suggesting that the omission predates AI research. Its importance nevertheless increases when the technology operates directly within the under-measured process.

### 5.7. Implications

For researchers, three priorities follow from the gaps identified above. The first is to import calibration paradigms into AI-mediated L2 designs: judgements of learning collected before and after AI-supported tasks and compared against blinded performance, together with indices of bias and resolution (Nelson & Narens, 1990). Instruments for this exist and require no new technology. The second is to triangulate self-report with at least one process modality. Generative platforms log prompt sequences, revision histories, and interaction timing as a by-product of use, which makes such data cheaper to collect than in any previous technological era; Yan and Zhang (2024) and Lai et al. (2026) demonstrate what becomes visible when it is collected. The third is to report metacognitive facets separately rather than as composite self-regulated-learning scores. The conflation of metacognition with self-regulated learning that Dinsmore et al. (2008) documented persists in this literature, and it obscures precisely the distinctions—between knowing, regulating, and judging accurately—that the present technology places under pressure.

For teachers, the finding that diagnostic and generative functions co-occur in 27% of post-ChatGPT tools, and that 41% carry two or more orientations, has a practical consequence. Institutional policies framed only in terms of permitting or prohibiting particular AI tools overlook variation in how the same tool is used: one prompt may elicit diagnosis, whereas the next may delegate the task itself. Instruction should therefore target prompting decisions themselves, which is where metacognitive control now operates. M.-R. A. Chen (2024) offers one worked example, in which metalinguistic guidance was embedded so that the system responded to learner analysis rather than replacing it, and Lukešová and Jennings (2026) another, in which learners received clues before corrections. X. Xu et al. (2025) similarly report that metacognitive support embedded in generative-AI environments is what distinguishes productive from unproductive use.

For tool designers, the evidence is consistent with building friction into interfaces where substitution is possible: defaulting to critique before draft, displaying the learner’s prior attempt alongside generated text, or requiring an explicit request before target-language output is produced. These are hypotheses for experimental testing, not conclusions this review can support.

For authors and journals, we propose a modest reporting convention. Where metacognition is named in a title or abstract, authors should indicate whether it was measured or served as a framing construct. Contextual and framing uses of a construct are legitimate, but the two roles are indistinguishable to a reader who screens titles and abstracts, and reviews built on such screening absorb studies in which the construct is named but never assessed. Indicating the distinction in the abstract would remove this source of error at negligible cost.

### 5.8. Limitations

Several limitations bound these claims. First, the review was not registered for the reason given in Section 3.1. Although a protocol and dated amendment log were prepared prospectively, registration would have provided stronger protection against selective reporting; moreover, the dimension that initially supported the configurational analysis was added after the protocol. Second, the search covered six databases on three platforms but excluded specialist indexes (LLBA, ACM Digital Library, and IEEE Xplore) and conference literature, potentially underrepresenting technical work from the neural era. Differences in field coverage across platforms may also have affected recall. Third, the scripted triage was directly validated as described in Section 3.4. It recovered two eligible studies and none from either sampled stratum, but the resulting false-negative rate remains bounded rather than zero. Fourth, backward and forward citation searching, together with a supplementary search using a broader population module, identified 38 additional candidate records; eleven appear eligible and six require full-text assessment. These records were excluded because the corpus was defined by the three database families in Section 3.2 and are reported only as a sensitivity analysis. The supplementary search exposed a narrowness in the population module, which combined L2, second language, foreign language, EFL, ESL and TESOL but omitted English for academic purposes, English academic writing, interpreting and CLIL; eligible work described in those terms was retrievable only by other means. They do not alter the conclusions: only one of the 38 mentions a calibration-related index, and that record is neither L2-specific nor quantitative on this issue. Fifth, the included literature is geographically concentrated and dominated by higher-education EFL research, partly because of source coverage. Against a baseline of AI and L2 learning publications in the same window (OpenAlex, journal articles, three-module match, *n* = 3835), first authors based in China, including Hong Kong, Macau and Taiwan, are over-represented by 25 percentage points, while Indonesia, the largest single contributor to that baseline at 17%, supplies 3% of the corpus; four of the candidate records identified by the supplementary search appear in Indonesian journals, suggesting indexing rather than absence. The comparison table appears in Supplementary File S6. Sixth, the neural-era group contains only four studies, precluding statistical tests of generational differences. Finally, the locus dimension was difficult to code reliably until admissible evidence was restricted to design descriptions. The recoded dimension is therefore reported descriptively and does not support causal inference.

## 6. Conclusions

Metacognition is central to second and foreign language learning because it enables learners to plan, monitor, and evaluate their comprehension and production. Decades of L2 research have established both the theoretical foundations and the methods required to investigate these processes (Flavell, 1979; Veenman et al., 2006). AI places this capacity under new pressure by performing regulatory work that learners previously carried out themselves. Earlier systems externalised specific functions such as diagnosis and evaluation, whereas generative AI can participate in planning, drafting, revision, and feedback. Whether this support strengthens learners’ regulatory capacity or substitutes for its development remains an empirical question with direct implications for language learning and instructional design.

This review synthesised sixty studies of metacognition in AI-supported L2 learning published between 2016 and May 2026, coding fourteen analytical dimensions through double-coded procedures and criterion-level quality appraisal. Three findings stand out. First, the evidence is overwhelmingly recent: only four eligible studies preceded the public release of ChatGPT, whereas fifty-six followed it. Second, generative systems increasingly combine functions that earlier tools kept separate. In 41% of post-ChatGPT studies, tools combined two or more functional orientations within one conversational interface; diagnostic and generative functions co-occurred in 27%. Traditional classifications of AI applications do not adequately represent this configuration because the same interface can guide the learner or produce the product, depending largely on the learner’s request. Third, the evidence relies heavily on self-reports of metacognitive regulation. Regulation was measured in 98% of post-ChatGPT studies, 66% relied exclusively on self-report, and only one study in the full corpus examined whether learners’ judgements corresponded to their demonstrated competence.

A fourth pattern requires separate treatment because its evidential status differs. The dimension identifying who executes the meta-level operation was added after the protocol, and a provenance audit conducted in response to review found that the two cases supporting the previously reported association had been coded from study findings rather than from descriptions of AI deployment. Recoding under an explicit provenance rule increased agreement between the two authors from κ = 0.32 in the 26-study blind pilot to κ = 0.71, and to κ = 0.90 for the 18 studies retained after full-text re-inspection of design. The recoding also emptied the substitution category: none of the 18 contrast-based studies assigned the learner’s regulatory operation to the system. The previously reported association is therefore withdrawn. The remaining finding is structural: the condition identified by theory as most consequential has not been tested, and related interface recommendations remain hypotheses for future experiments rather than conclusions supported by this review.

These findings identify four methodological priorities. First, calibration paradigms should compare judgements of learning before and after AI-supported tasks with blinded measures of performance. The single study that used this approach provides a workable template by pairing item-level judgements with performance on the same items and reporting bias, error, discrimination, and a calibration curve. Second, self-report should be complemented by process evidence, including interaction logs, revision histories, prompted think-aloud protocols, and trace data already produced by generative platforms. Third, studies should report metacognitive components separately rather than collapsing them into broad composite scores, allowing the effects on planning, monitoring, evaluation, and metacognitive knowledge to be distinguished. Fourth, the evidence base should extend beyond higher-education EFL populations to younger learners, multilingual contexts, and longitudinal designs capable of capturing developmental change.

The findings also sharpen the theoretical agenda. Research should identify which metacognitive functions AI scaffolds, which it replaces, and the instructional and learner conditions associated with each outcome (Chang & Sun, 2024). It must also distinguish regulation from calibration because effective regulation does not guarantee accurate judgements of one’s knowledge or performance (Flavell, 1979; Veenman et al., 2006). Learners may complete AI-supported tasks more efficiently while becoming less accurate about what they can accomplish independently, a possibility suggested by recent evidence (Y. Fan et al., 2025). Testing whether AI promotes reflective regulation, facilitates offloading, or produces miscalibrated self-assessment requires researchers to model explicitly how regulatory responsibility is distributed between learners and AI systems (Chang & Sun, 2024; Risko & Gilbert, 2016).

As generative systems become more capable, learners may find it increasingly difficult to distinguish assisted performance from independent competence. The central metacognitive question is therefore not only whether learners regulate their learning, but whether they can accurately identify what they know, what the system contributes, and what the learner–AI partnership can accomplish. Without calibration measures, studies risk conflating system-supported performance with learner competence (Veenman et al., 2006). The necessary measurement methods already exist. Applying them systematically to AI-mediated language learning would allow the field to determine who performs the regulatory work and whether learners accurately perceive that division of responsibility.

## Figures and Tables

**Table 1:** Coding scheme, categories, and taxonomic sources.

Dimension	Categories	Source
D1 Era	Era 1, neural AI (January 2016–November 2022); Era 2, post-ChatGPT (December 2022–May 2026)	Dated by earliest online availability; era boundary set at the public release of ChatGPT
D2a AI application type	Profiling and prediction; assessment and evaluation; adaptive systems and personalisation; intelligent tutoring systems; content generation and general-purpose assistants	Zawacki-Richter et al. (2019), applied to L2 by S. Qiao et al. (2025); fifth category added in this review
D2b AI functional orientation (multi-label)	Diagnostic (AI evaluates learner output); generative (AI performs work the learner would otherwise do); dialogic (AI elicits the learner’s reasoning)	Derived from the monitoring–control architecture of Nelson and Narens (1990)
D3 Metacognitive facet (multi-label)	Metacognitive knowledge; metacognitive regulation; metacognitive experiences; metacognitive accuracy (calibration)	Flavell (1979); Brown (1987); Schraw and Dennison (1994); Efklides (2011); Nelson and Narens (1990)
D4 Role of metacognition in the design	Outcome of AI use; guiding framework for design; strategic process during AI use	Transposed from the three SRL–AI relationships in Z. Zhang et al. (2025)
D5 Measurement modality (multi-label)	Offline self-report; online process data (counted only when analysed as metacognitive evidence)	Offline–online distinction of Veenman et al. (2006)
D6 Direction of evidence	Positive; negative; null; mixed; not applicable; no effect estimated	Judged on the AI-to-metacognition path only; stratified by design-based evidence stratum (Section 3.7)
D7 Educational level (multi-label)	Elementary; junior secondary; senior secondary; higher education; adult or vocational	Hwang and Tsai (2011), extended with an adult or vocational category
D8 Research design	First order: quantitative, qualitative, blended. Second order: survey, correlational, causal-comparative, experimental, single-subject	Gay et al. (2009)
D9 Language skill or content domain (multi-label)	Listening; speaking and pronunciation; reading; writing; vocabulary; grammar; translation; integrated or general proficiency	Skill organisation of Golonka et al. (2014), cross-checked against the domain categories of S. Qiao et al. (2025) and Y. Wang et al. (2026)
D10 Learning-theoretical orientation (multi-label)	Behaviourism; cognitivism; constructivism; sociocultural; other named theory; none stated	Three paradigms adopted from Ertmer and Newby (1993), extended inductively with a sociocultural category, a residual category for other named theories, and a “none stated” category
D11 Learner proficiency and target language	Beginner (A1–A2); intermediate (B1–B2); advanced (C1+); mixed; not reported/target language as reported	CEFR bands (Council of Europe, 2001)
D12 Metacognitive regulation sub-process (multi-label)	Planning; monitoring; evaluation; strategy regulation; undifferentiated composite	Brown (1987); regulation subscales of Schraw and Dennison (1994)
D13 Locus of the meta-level operation	Scaffold (AI prompts, learner executes); transformation (system executes part; learner judges whether to adopt); substitute (AI executes and delivers); not determinable	Derived inductively; judged by the meta-level–object-level architecture of Nelson and Narens (1990)
D14 Instructional conditions	Explicit metacognitive scaffolding (yes/no); duration (single session, <4, 4–8, >8 weeks, not reported); guidance (teacher-guided, learner-autonomous, mixed, not reported)	Condition set coded for the descriptive cross-tabulation reported in subsequent tables; the necessity analysis originally planned following Thomas et al. (2014) was replaced because the substitution category is empty

**Table 2:** Direction of reported metacognitive evidence by design-based evidence stratum (*N* = 60).

Evidence Stratum	n/a	No Effect Estimated	Positive	Mixed	Negative	Null
S1 Contrast-based	1	0	13	3	1	0
S2 Single-group/case	1	0	3	2	1	0
S3 Correlational/descriptive	5	4	11	3	2	1
S4 Qualitative	0	0	9	0	0	0

Note. Cell entries are counts of studies. n/a = no direction of evidence could be coded because the study reported no metacognitive outcome measure; No Effect Estimated = scale-development or validation studies, which estimate no effect; Null = a metacognitive outcome was measured and no difference was detected.

**Table 3:** Direction of reported metacognitive evidence by instructional condition (contrast-based studies, *n* = 18).

Condition	*n*	Positive	Mixed	Negative	Null
AI as scaffold	5	3	1	0	0
AI as transformation	10	7	2	1	0
AI as substitute	0	0	0	0	0
Locus not determinable	3	3	0	0	0
Explicit metacognitive scaffolding embedded	11	8	2	0	0
No explicit metacognitive scaffolding	7	5	1	1	0
AI exposure > 8 weeks	13	10	1	1	0
AI exposure 4–8 weeks	5	3	2	0	0
Teacher-guided use	13	10	2	1	0
Mixed guidance	3	2	1	0	0
Learner-autonomous use	2	1	0	0	0

Note. Cell entries are counts of contrast-based studies. Conditions within a block (locus; explicit metacognitive scaffolding; AI-exposure duration; guidance) are mutually exclusive.

## Data Availability

All data supporting the reported results are contained within the article and its Supplementary Materials. The complete record-level coding tables (Files S4 and S5), the search strategies (File S1), and the search-recall validation workbook (File S6) document every judgement on which the reported counts are based. No new primary data were created.

## Supplementary Materials

The following supporting information can be downloaded at: https://www.mdpi.com/article/10.3390/jintelligence14100236/s1, Supplementary File S1. Database search strategies and platform-specific syntax. Supplementary File S2. Prospective protocol and dated amendment log. Supplementary File S3. PRISMA 2020 checklists (main and abstract). Supplementary File S4. Complete coding table for the eight protocol dimensions. Supplementary File S5. Complete coding table for the dimensions added. Supplementary File S6. Search-recall validation workbook.

## Appendix A

**Table A1:** MMAT 2018 criterion-level appraisal of the 60 included studies (✓ = yes, ✗ = no, ? = cannot tell). No overall score is computed, following the guidance of the instrument’s developers.

Study	Year	Era	Design	S1	S2	Q1	Q2	Q3	Q4	Q5
A14	2019	Era1	NONRAND	✓	✓	?	?	✗	✗	?
A18	2020	Era1	DESCRIPTIVE	✓	✓	✓	✗	✓	?	✓
A08	2021	Era1	QUAL	✓	✓	✓	✓	✓	✓	✓
A05	2022	Era1	RCT	✓	✓	?	✓	✓	✗	✓
C02	2023	Era2	RCT	✓	✓	?	✓	✓	✗	?
E31	2023	Era2	NONRAND	✓	✓	?	?	✓	✓	✓
C29	2024	Era2	DESCRIPTIVE	✓	?	?	✗	?	?	✗
D02	2024	Era2	NONRAND	✓	✓	?	✓	✓	✓	✓
D06	2024	Era2	MIXED	✓	✓	✓	✓	✓	?	✗
D08	2024	Era2	MIXED	✓	✓	✓	✓	✓	?	✗
D14	2024	Era2	MIXED	✓	✓	✓	✓	✓	?	✗
D18	2024	Era2	QUAL	✓	✓	✓	✓	✓	✓	✓
D39	2024	Era2	MIXED	✓	✓	?	?	?	✓	✗
E16	2024	Era2	DESCRIPTIVE	✓	✓	✓	✗	✓	?	✓
C10	2025	Era2	MIXED	✓	✓	✓	✓	✓	✓	✗
C15	2025	Era2	MIXED	✓	✓	✓	✓	?	✓	✗
C20	2025	Era2	QUAL	✓	✓	✓	✓	✓	✓	✓
C37	2025	Era2	MIXED	✓	✓	?	?	✓	?	✗
D07	2025	Era2	MIXED	✓	✓	✓	✓	✓	?	?
D15	2025	Era2	MIXED	✓	✓	?	✓	✓	✓	✗
D17	2025	Era2	MIXED	✓	✓	✓	?	?	?	?
D19	2025	Era2	MIXED	✓	✓	✓	✓	✓	?	✓
D22	2025	Era2	MIXED	✓	✓	✓	✓	✓	?	✗
D27	2025	Era2	DESCRIPTIVE	✓	?	?	✗	✗	?	✗
D37	2025	Era2	MIXED	✓	✓	✓	✓	✓	?	✗
D48	2025	Era2	QUAL	✓	✓	✓	✓	✓	✓	✓
D55	2025	Era2	DESCRIPTIVE	✓	✓	?	✗	✓	?	?
D63	2025	Era2	RCT	✓	✓	?	✓	✓	?	?
E05	2025	Era2	DESCRIPTIVE	✓	✓	✓	?	✓	✓	✓
E12	2025	Era2	QUAL	?	?	✓	?	?	?	✗
E15	2025	Era2	MIXED	✓	✓	✓	✓	✓	✓	✗
E20	2025	Era2	NONRAND	✓	✓	✗	✓	✓	✗	?
E25	2025	Era2	QUAL	✓	✓	✓	✓	✓	✓	✓
E28	2025	Era2	MIXED	✓	✓	✗	✓	?	?	✗
C03	2026	Era2	RCT	✓	✓	✓	?	✓	✓	?
C07	2026	Era2	MIXED	✓	✓	✓	✓	✓	✓	✗
C11	2026	Era2	MIXED	✓	✓	✓	✓	✓	?	?
C17	2026	Era2	QUAL	✓	✓	✓	✓	✓	✓	✓
C21	2026	Era2	MIXED	✓	✓	✓	✓	✓	✓	✗
D03	2026	Era2	MIXED	✓	✓	✗	✓	✓	?	?
D04	2026	Era2	DESCRIPTIVE	✓	✓	✓	?	✓	✓	✓
D05	2026	Era2	MIXED	✓	✓	✓	✓	✓	✓	?
D10	2026	Era2	MIXED	✓	✓	✓	✓	✓	✓	✓
D11	2026	Era2	DESCRIPTIVE	✓	✓	?	✗	✓	?	✓
D16	2026	Era2	QUAL	✓	✓	✓	✓	✓	✓	✓
D21	2026	Era2	MIXED	✓	✓	✓	✓	✓	?	✓
D25	2026	Era2	DESCRIPTIVE	✓	✓	✓	✗	✓	?	?
D28	2026	Era2	MIXED	✓	✓	✓	✓	✓	?	✗
D29	2026	Era2	QUAL	✓	✓	✓	✓	✓	?	✓
D46	2026	Era2	MIXED	✓	✓	✓	✓	✓	?	?
D47	2026	Era2	DESCRIPTIVE	✓	✓	✓	?	✓	?	✓
D49	2026	Era2	MIXED	✓	✓	✓	?	✓	✓	✗
D53	2026	Era2	NONRAND	✓	✓	✗	✓	✓	✗	✓
D56	2026	Era2	MIXED	✓	✓	✓	✓	✓	✓	?
E02	2026	Era2	DESCRIPTIVE	✓	✓	✓	?	✓	✓	✓
E04	2026	Era2	NONRAND	✓	✓	?	✓	✓	✓	✓
E21	2026	Era2	DESCRIPTIVE	✓	✓	?	✗	?	?	✓
E23	2026	Era2	DESCRIPTIVE	✓	✓	?	?	✓	?	✓
C40	2025	Era2	MIXED	✓	✓	✓	✓	?	?	✓
D64	2026	Era2	DESCRIPTIVE	✓	✓	✓	?	✓	?	✓

## Appendix B

**Table A2:** Overview of the 60 included studies. Entries marked “uncertain” retained a residual coding uncertainty after adjudication and were counted under the category shown.

ID	Year (Online-First)	Era	Journal	AI Application	Function (s)	Direction	Stratum
A14	2019	Era1	Computer Assisted Language Learning	Intelligent tutoring systems	Diagnostic	n/a	S1
A18	2020	Era1	Interactive Learning Environments	Content generation and general-purpose assistants	Generative	n/a	S3
A08	2021	Era1	Computer Assisted Language Learning	Adaptive systems and personalisation	Diagnostic	Positive	S4
A05	2022	Era1	Computer Assisted Language Learning	Assessment and evaluation	Diagnostic	Mixed	S1
C02	2023	Era2	Frontiers in Psychology	Adaptive systems and personalisation	Diagnostic; Dialogic	Positive	S1
E31	2023	Era2	Interactive Learning Environments	Content generation and general-purpose assistants	Generative (uncertain)	Mixed	S3
D08	2024	Era2	European Journal of Education	Assessment and evaluation	Diagnostic; Generative	Positive	S1
D18	2024	Era2	ELT Journal	Assessment and evaluation	Diagnostic	Positive	S4
D06	2024	Era2	Journal of Computer Assisted Learning	Content generation and general-purpose assistants	Diagnostic; Generative	Mixed	S3
E16	2024	Era2	International Journal of TESOL Studies	Content generation and general-purpose assistants	Generative	n/a	S3
D14	2024	Era2	Humanities & Social Sciences Communications	Assessment and evaluation	Diagnostic	Negative	S2
D39	2024	Era2	English Teaching (South Korea)	Content generation and general-purpose assistants	Dialogic	Positive	S3
D02	2024	Era2	Educational Technology & Society	Assessment and evaluation	Diagnostic	Positive	S3
C29	2024	Era2	Theory and Practice in Language Studies	Assessment and evaluation	Diagnostic	Positive	S3
C20	2025	Era2	TEFLIN Journal	Content generation and general-purpose assistants	Generative	Positive	S4
E25	2025	Era2	Journal of Pedagogical Research	Assessment and evaluation (uncertain)	Diagnostic	Positive	S4
C10	2025	Era2	Education and Information Technologies	Assessment and evaluation	Diagnostic; Dialogic	Positive	S1
E15	2025	Era2	Discover Artificial Intelligence	Adaptive systems and personalisation	Dialogic	Mixed	S1
D48	2025	Era2	Journal of Asia TEFL	Assessment and evaluation	Diagnostic; Generative; Dialogic	Positive	S4
D15	2025	Era2	System	Assessment and evaluation	Diagnostic	Positive	S3
E05	2025	Era2	Assessment & Evaluation in Higher Education	Assessment and evaluation	Diagnostic	Positive	S3
E28	2025	Era2	Indonesian Journal of English Language Teaching and Applied Linguistics	Assessment and evaluation	Diagnostic; Generative	Positive	S3
D19	2025	Era2	Journal of Computer Assisted Learning	Assessment and evaluation	Diagnostic; Generative	Positive	S1
D07	2025	Era2	British Journal of Educational Technology	Content generation and general-purpose assistants	Diagnostic; Generative	Positive	S1
D55	2025	Era2	TEFLIN Journal	Content generation and general-purpose assistants	Generative	No effect estimated	S3
D63	2025	Era2	Language Teaching Research	Content generation and general-purpose assistants	Diagnostic; Generative	Positive	S1
D22	2025	Era2	Innovation in Language Learning and Teaching	Assessment and evaluation	Diagnostic	Mixed	S2
D37	2025	Era2	Edelweiss Applied Science and Technology	Intelligent tutoring systems	Diagnostic; Dialogic	Positive	S2
C37	2025	Era2	SAGE Open	Profiling and prediction	Diagnostic	Positive	S2
D17	2025	Era2	System	Assessment and evaluation	Diagnostic; Generative	No effect estimated	S3
C15	2025	Era2	International Journal of Learning, Teaching and Educational Research	Assessment and evaluation	Diagnostic	Mixed	S2
D27	2025	Era2	International Journal of Applied Linguistics	Content generation and general-purpose assistants	Diagnostic; Generative	n/a	S3
E12	2025	Era2	Dirasat: Human and Social Sciences	Adaptive systems and personalisation	Diagnostic	Positive	S4
E20	2025	Era2	Computers and Education: Artificial Intelligence	Assessment and evaluation	Diagnostic; Dialogic	n/a	S2
D04	2026	Era2	Acta Psychologica	Assessment and evaluation	Diagnostic	Positive	S3
D53	2026	Era2	Frontiers in Education	Intelligent tutoring systems	Dialogic	Positive	S1
D05	2026	Era2	Computer Assisted Language Learning	Content generation and general-purpose assistants	Diagnostic; Generative	Negative	S3
C03	2026	Era2	Language Teaching Research	Assessment and evaluation	Diagnostic	Positive	S1
E23	2026	Era2	TESL-EJ	Content generation and general-purpose assistants	Diagnostic; Generative	n/a	S3
C21	2026	Era2	World Journal of English Language	Assessment and evaluation	Diagnostic	Negative	S1
D47	2026	Era2	International Journal of Learning, Teaching and Educational Research	Content generation and general-purpose assistants	Generative	Positive	S3
D46	2026	Era2	Arab World English Journal	Content generation and general-purpose assistants	Generative	Positive	S1
C17	2026	Era2	Cogent Education	Assessment and evaluation	Diagnostic	Positive	S4
E04	2026	Era2	Journal of English for Academic Purposes	Assessment and evaluation	Diagnostic; Dialogic	Positive	S1
E02	2026	Era2	Acta Psychologica	Intelligent tutoring systems	Diagnostic; Dialogic	Positive	S3
D49	2026	Era2	Digital Technologies Research and Applications	Content generation and general-purpose assistants	Diagnostic; Generative	Negative	S3
D56	2026	Era2	Cogent Education	Assessment and evaluation	Diagnostic; Generative	Positive	S1
D29	2026	Era2	Innovation in Language Learning and Teaching	Assessment and evaluation	Dialogic	Positive	S4
D11	2026	Era2	IRAL—International Review of Applied Linguistics in Language Teaching	Content generation and general-purpose assistants	Generative	No effect estimated	S3
C11	2026	Era2	Acta Psychologica	Adaptive systems and personalisation	Diagnostic	Positive	S3
C07	2026	Era2	Australasian Journal of Educational Technology	Assessment and evaluation	Diagnostic; Dialogic	Positive	S1
D16	2026	Era2	Frontiers in Psychology	Content generation and general-purpose assistants	Generative	Positive	S4
E21	2026	Era2	Asian Journal of English Language Teaching	Content generation and general-purpose assistants	Diagnostic; Generative; Dialogic	No effect estimated	S3
D03	2026	Era2	Assessing Writing	Assessment and evaluation	Diagnostic	Positive	S3
D21	2026	Era2	Discover Computing	Content generation and general-purpose assistants	Generative; Dialogic	Mixed	S3
D10	2026	Era2	Computers & Education	Content generation and general-purpose assistants	Generative; Diagnostic	Mixed	S1
D25	2026	Era2	Asia-Pacific Education Researcher	Content generation and general-purpose assistants	Generative	Null	S3
D28	2026	Era2	Acta Psychologica	Assessment and evaluation	Diagnostic	Positive	S2
C40	2025	Era2	Environment and Social Psychology	Assessment and evaluation	Diagnostic	Positive	S1
D64	2026	Era2	International Journal of Applied Linguistics	Content generation and general-purpose assistants	Diagnostic	n/a	S3
