# Supplementary material for: Metacognition in AI-Supported Second Language Learning: A Systematic Review of Constructs, Measures, and Evidence (2016–2026)
Source: J Intell. 2026 Oct 1;14(10):236. doi: 10.3390/jintelligence14100236 (PMC13706126; doi:10.3390/jintelligence14100236)
Supplement: Supplementary file 1 [file jintelligence-14-00236-s001.zip › Supplementary_S1_Search_strategies.pdf]

Supplementary S1. Full search strategies and AI-mediation keyword list

S1.1 Web of Science Core Collection (searched June 2026, updated 7 August 2026; SSCI, SCIE, A&HCI, CPCI-S, CPCI-SSH, ESCI; conference proceedings retrieved through CPCI-S and CPCI-SSH were excluded under the document-type rule, S2 amendment 4)

```
TS=(metacogniti* OR meta-cogni* OR "meta cognit*") AND TS=(L2 OR
"second language" OR "foreign language" OR EFL OR ESL OR TESOL) AND
AB=(finding OR result OR experiment* OR control OR intervent*);limited
to English, Article
```

S1.2 Scopus (searched June 2026, updated 7 August 2026)

```
TITLE-ABS-KEY(metacogniti* OR meta-cogni* OR "meta cognit*") AND TITLE-
ABS-KEY(L2 OR "second language" OR "foreign language" OR EFL OR ESL OR
TESOL) AND TITLE-ABS-KEY(finding OR result OR experiment* OR control OR
intervent*); limited to English, Article
```

S1.3 EBSCOhost: Academic Search Complete, ERIC, APA PsycINFO, Education Source (searched June 2026, updated 7 August 2026)

```
SU(metacogniti* OR meta-cogni* OR "meta cognit*") AND SU(L2 OR "second
language" OR "foreign language" OR EFL OR ESL OR TESOL) AND AB(finding
OR result OR experiment* OR control OR intervent*); limited to English,
Academic Journals, Peer-reviewed
```

```
The empirical filter (finding OR result OR experiment* OR control OR
intervent*) is drawn from Guan et al. (2024). In Web of Science and on
EBSCOhost it is applied to the abstract field; in Scopus, to title-
abstract-keyword fields.
```

S1.4 Positive-control and recall validation

Four articles known a priori to meet all criteria were checked against each export. Web of Science 4/4; Scopus 4/4; EBSCOhost 3/4 (the miss traced to missing abstract metadata in the source record). After peer review the recall of the scripted triage was validated directly: all 2,581 records the script had excluded were re-screened with a deliberately over-inclusive lexicon that strictly contains the one used here, which recalled 184/184 of the candidate records the original procedure had retained; 543 of the excluded records (21.0%) were then screened manually as a census of the flagged stratum plus random samples of the other two. Two eligible studies were recovered. The full workbook is Supplementary S6. A CrossRef-based supplementary search was conducted on 7 August 2026 as the update search.

S1.5 AI-mediation keyword list (bibliometric layer flag and screening triage)

```
chatgpt; gpt-3/4/5; generative AI/artificial intelligence; large
language model; LLM; gemini; copilot; deepseek; claude; bard; ai-
generated; ai chatbot; ai writing assistant; intelligent tutor(ing);
ICALL; automated writing evaluation; automated essay scoring; automated
(corrective) feedback; automatic speech recognition; ASR; pronunciation
training; CAPT; adaptive learning/system; recommender system;
conversational agent; dialogue system; pedagogical agent; chatbot;
```

voice assistant; machine translation; NMT; natural language processing; NLP; machine learning; deep learning; neural network; learning analytics; educational data mining; named tools: Duolingo, Grammarly, Criterion, Pigai, Write & Improve, DeepL, Google Translate, Papago, Youdao, ELSA Speak, Speechace, Liulishuo, Replika; ai-driven/-enhanced/-supported/-assisted/-mediated/-powered/-based
