# Supplementary material for: Metacognition in AI-Supported Second Language Learning: A Systematic Review of Constructs, Measures, and Evidence (2016–2026)
Source: J Intell. 2026 Oct 1;14(10):236. doi: 10.3390/jintelligence14100236 (PMC13706126; doi:10.3390/jintelligence14100236)
Supplement: Supplementary file 1 [file jintelligence-14-00236-s001.zip › Supplementary_S2_Protocol_and_amendments.pdf]

Supplementary S2. Review protocol and amendment log

This review was not registered. PROSPERO does not accept reviews without a health-related outcome, and no equivalent register was available for this topic. A protocol was written prospectively, before screening and coding began, and was held fixed thereafter. The protocol was drafted in Chinese as a working document; what follows is a faithful English rendering of its substantive provisions. Internal notes on target-journal formatting, self-citation ratios and project scheduling have been omitted as they form no part of the review method. Every departure from the protocol, whether decided during the review or during revision, is listed in the amendment log in Part B.

Part A. Protocol

A1. Objective and construct boundary

The review examines how metacognition has been conceptualised and measured in second and foreign language (L2) learning under AI mediation, and how that changed across two adjacent technological generations. The construct boundary was fixed as strict metacognition rather than the whole of self-regulated learning (SRL). The protocol records the direction of the relation explicitly: SRL is not a subordinate of metacognition; in the mainstream position metacognition is one component of SRL (Zimmerman; Pintrich; Dinsmore, Alexander, & Loughlin, 2008; Panadero, 2017). The review therefore narrows deliberately, and the protocol requires that this narrowing be stated in the Introduction.

A2. Theoretical framework

A two-layer framework was fixed. Layer one is the meta-level/object-level architecture of Nelson and Narens (1990), distinguishing monitoring (object level to meta level: judgements of learning, feeling of knowing, self-assessment, calibration) from control (meta level to object level: study-time allocation, restudy decisions, strategy selection). Layer two is Flavell's (1979) tripartite division of metacognitive knowledge into person, task and strategy knowledge. The protocol prohibits the use of the COPES architecture, to keep the review distinct from existing SRL syntheses.

The protocol states one falsifiable core claim: pre-generative technologies (ITS, AWE, ASR, adaptive systems) externalise the monitoring step through diagnostic feedback, which learners may internalise as improved calibration, whereas generative AI severs the control step by substituting for the object-level product, leaving monitoring without an object. Four testable generational expectations were derived from it, concerning calibration direction, which knowledge node is affected, migration of measurement modality, and whether observed generational differences are driven by technology or by shifts in research fashion.

A3. Four facets of metacognition (operational inclusion list)

- Metacognitive knowledge: person, task and strategy knowledge (Flavell, 1979; Schraw & Dennison, 1994; Wenden, 1998).
- Metacognitive regulation: planning, monitoring, evaluating, plus information management and debugging (Brown, 1987; Schraw & Dennison, 1994).
- Metacognitive experiences: feeling of knowing, feeling of difficulty, confidence, judgements of learning, effort and time estimates (Flavell, 1979; Efklides, 2011).

- Metacognitive accuracy and calibration: bias (absolute accuracy), resolution or gamma (relative accuracy), sensitivity, overconfidence, the illusion of competence (Nelson & Narens, 1990; Veenman et al., 2006).

The protocol identifies the fourth facet as the point of differentiation from existing reviews, none of which addresses it.

A4. Exclusion list for adjacent constructs

Excluded outright: motivation, self-efficacy, goal orientation, task value; emotion regulation and anxiety; time management, environment structuring and effort regulation; acceptance intention and technology acceptance measures; language achievement or writing quality without any metacognitive measure; teacher rather than learner metacognition unless learner metacognition is also reported. Admitted conditionally, by independent adjudication of two coders: learner autonomy only when operationalised as planning, monitoring or evaluating; critical thinking only when the measure contains a self-regulation or self-evaluation dimension; help-seeking only when monitoring-driven and argued by the authors as metacognitive control; reflection only when its object is the learner's own cognition; cognitive offloading only when a metacognitive consequence is the outcome variable; feedback literacy only when it contains self-evaluative judgement.

A5. Generational design and dating rule

The cut point is 30 November 2022, the public release of ChatGPT. Era 1 (neural AI) runs from the start of the window to that date; Era 2 (post-ChatGPT) from 1 December 2022. Studies in Era 2 need not have used generative AI, and "generative AI studies" is reserved for studies that used generative functionality. Studies are dated by earliest public availability, taking CrossRef published-online in preference to published-print, and not by issue year. The protocol records that database year fields carry the issue year and that this misassigns cases; sixteen candidate records changed era assignment under the rule.

Two boundary rules were fixed after the first live screening pass and before full-text coding. Rule B1: the technology must mediate learning, standing between the learner and the language task, and not merely serve as a research instrument. Rule B2: where an article names no AI type, the study must not be assigned by guesswork; it goes to full-text identification, and if still undetermined it is recorded as unspecified and reported as such.

A6. Eligibility criteria

Population: learners of a second or foreign language at any educational level and in any target language. Intervention: intervention with, use of, or exposure to an AI technology. Outcome: at least one explicit metacognitive dependent or process variable, conceptualised by the authors as metacognition or measured with a recognised metacognitive instrument. Study type: empirical research, quantitative, qualitative or mixed, reporting extractable results. Excluded: studies reporting only an SRL total with no separable metacognitive component; studies whose outcomes are only motivational, affective or behavioural; studies with no metacognitive measure.

A7. Screening, reliability and coding

Independent double screening, Cohen's κ computed at title-abstract and full-text stages, disagreements adjudicated by the third author with the number and type of disagreements recorded; PRISMA 2020

counts logged throughout with categorised full-text exclusion reasons. Pilot coding on a stratified random sample of 14 articles (19.7% of the full texts retrieved), with the coding form revised before full coding and coding κ reported. Quality appraisal with MMAT 2018.

A8. Synthesis strategy

The primary analysis is a configurative synthesis organised as an evidence map by generation and metacognitive sub-construct. Generational comparison proceeds by direction vote-counting within each sub-construct with descriptive comparison of effect sizes, without forced pooling. Meta-analysis is conditional and additional, to be attempted only where a sub-construct carries $k \geq 10$ in each generation with comparable indices; the protocol states in advance that the primary analysis is narrative, so that nothing is promised that cannot be delivered.

Part B. Amendment log

Amendments 1–9 were made before screening began, between the initial search (June 2026) and the CrossRef update search (7 August 2026). Amendments 10–14 were made during revision, in response to review.

#	Date	Amendment
1	—	Information sources: six databases on three platforms were searched.
2	—	Review window: 2016-01-01 to 2026-05-31.
3	—	Search string: the AI module was omitted at retrieval; an empirical filter (Guan et al., 2024) was applied on all three platforms (abstract field in Web of Science and on EBSCOhost; title–abstract–keyword fields in Scopus).
4	—	Document types: conference papers were excluded.
5	—	Eligibility: tightened to AI-mediated research.
6	—	Research questions: reduced from five to three.
7	—	Analytic design: reported as two technological eras.
8	—	Coding manual: adjudication-derived decision rules were added.
9	—	Coding dimensions: six dimensions (D9–D14) were added after the protocol.
10	2026-09-01	Evidence-provenance rule introduced for the regulatory-locus dimension.
11	2026-09-01	Corpus expanded from 58 to 60 studies.
12	2026-09-05	Necessity analysis replaced by a descriptive cross-tabulation.
13	2026-09-06	Two studies reclassified out of the contrast-based stratum after full-text re-inspection.
14	2026-09-06	Six further studies reclassified out of the stratum; the stratum now comprises 18 studies.
