# Supplementary material for: Metacognition in AI-Supported Second Language Learning: A Systematic Review of Constructs, Measures, and Evidence (2016–2026)
Source: J Intell. 2026 Oct 1;14(10):236. doi: 10.3390/jintelligence14100236 (PMC13706126; doi:10.3390/jintelligence14100236)
Supplement: Supplementary file 1 [file jintelligence-14-00236-s001.zip › Supplementary_S3_PRISMA2020_checklist.pdf]

Supplementary File S3

PRISMA 2020 Checklist

Metacognition in AI-Supported Second Language Learning: A Systematic Review of Constructs, Measures, and Evidence (2016–2026)

Submitted to Journal of Intelligence

Item wording follows Page, M. J., McKenzie, J. E., Bossuyt, P. M., Boutron, I., Hoffmann, T. C., Mulrow, C. D., et al. (2021). The PRISMA 2020 statement: An updated guideline for reporting systematic reviews. BMJ, 372, n71. https://doi.org/10.1136/bmj.n71. Locations refer to the revised manuscript; section numbers are those of the manuscript, and "S1–S6" refer to the supplementary files listed under Supplementary Materials.

Section and Topic	Item #	Checklist item	Location where item is reported
**TITLE**			
Title	1	Identify the report as a systematic review.	Title ("… A Systematic Review (2016–2026)").
**ABSTRACT**			
Abstract	2	See the PRISMA 2020 for Abstracts checklist.	Abstract; itemised in Table S3.2 below.
**INTRODUCTION**			
Rationale	3	Describe the rationale for the review in the context of existing knowledge.	§1; §2.1–§2.3.
Objectives	4	Provide an explicit statement of the objective(s) or question(s) the review addresses.	§2.4, final paragraph (RQ1–RQ3).
**METHODS**			
Eligibility criteria	5	Specify the inclusion and exclusion criteria for the review and how studies were grouped for the syntheses.	§3.3 (criteria a–d and exclusions); grouping by era (§3.3) and by design-based evidence stratum (§3.7).
Information sources	6	Specify all databases, registers, websites, organisations, reference lists and other sources searched or consulted to identify studies. Specify the date when each source was last searched or consulted.	§3.2: six databases on three platforms (Web of Science Core Collection; Scopus; and on EBSCOhost, Academic Search Complete, ERIC, APA PsycINFO, Education Source), searched June 2026 and updated by a CrossRef-based supplementary search on 7 August 2026. No registers, websites or organisations were consulted; backward and forward citation searching of the included studies and a supplementary search with a broadened population module are reported as a sensitivity analysis in §5.8.
Search strategy	7	Present the full search strategies for all databases, registers and websites, including any filters and limits used.	§3.2 (module structure and rationale for excluding AI terms); full platform-specific syntax, filters and limits in Supplementary S1.
Selection process	8	Specify the methods used to decide whether a study met the inclusion criteria of the review, including how many reviewers screened each record and each report retrieved, whether they worked independently, and if applicable, details of automation tools used in the process.	§3.4 (scripted keyword triage as an automation tool, its recall validated after review by re-screening all 2,581 excluded records against an expanded lexicon, with a census of the flagged stratum and random samples of the remainder; workbook in Supplementary S6) and §3.6 (all 71 retrieved full texts independently double-coded, with the coder arrangement stated).
Data collection process	9	Specify the methods used to collect data from reports, including how many reviewers collected data from each report, whether they worked independently, any processes for obtaining or confirming data from study	§3.5 (coding scheme, Table 1) and §3.6.

		investigators, and details of automation tools used in the process.	
Data items	10a	List and define all outcomes for which data were sought. Specify whether all results that were compatible with each outcome domain in each study were sought, and if not, the methods used to decide which results to collect.	§3.3 (criterion c: the four metacognitive facets) and §3.5 (D3, D12 and D6). All reported metacognitive results compatible with these facets were sought; where a study reported several, the direction code was assigned on the AI-to-metacognition path only (§3.5, Table 1).
Data items	10b	List and define all other variables for which data were sought. Describe any assumptions made about any missing or unclear information.	§3.5 and Table 1 (D1, D2a, D2b, D4, D5, D7–D14). Unclear information was coded "not reported" or, where a judgement remained unresolved after adjudication, flagged as uncertain and counted under the category shown (Table B1 note).
Study risk of bias assessment	11	Specify the methods used to assess risk of bias in the included studies, including details of the tool(s) used, how many reviewers assessed each study and whether they worked independently, and details of any automation tools used in the process.	§3.6, final paragraph: MMAT 2018 (Hong et al., 2018), design-specific branch plus two screening items, double-appraised by the two coders described in §3.6 working independently, with adjudication of disagreements by the third author. Full appraisal grid in Table A1.
Effect measures	12	Specify for each outcome the effect measure(s) (e.g., risk ratio, mean difference) used in the synthesis or presentation of results.	§3.7: no effect measure was synthesised. Results are presented as counts of studies by direction of evidence (positive, mixed, negative, null, not applicable) stratified by design-based evidence stratum (S1–S4).
Synthesis methods	13a	Describe the processes used to decide which studies were eligible for each synthesis (e.g., tabulating the study intervention characteristics and comparing against the planned groups for each synthesis).	§3.7 and §4.5: syntheses are tabulated by era (§4.1), by AI application type and functional orientation (§4.2), by metacognitive facet and modality (§4.3), by direction and design-based evidence stratum (§4.4, Table 2), and by instructional condition (§4.5, Table 3).
Synthesis methods	13b	Describe any methods required to prepare the data for presentation or synthesis, such as handling of missing summary statistics, or data conversions.	§3.5–§3.7. Multi-label dimensions are reported as counts of studies displaying each label, so percentages within a multi-label dimension exceed 100%; this is stated in the relevant figure captions. Instructional conditions were coded as described in §4.5 and cross-tabulated against the direction of evidence (Table 3); no necessity analysis is reported.
Synthesis methods	13c	Describe any methods used to tabulate or visually display results of individual studies and syntheses.	Figures 2–9 and Tables 2–3; study-level codings in Table B1 and Supplementary S4–S5.
Synthesis methods	13d	Describe any methods used to synthesise results and provide a rationale for the choice(s). If meta-analysis was performed, describe the model(s), method(s) to identify the presence and extent of statistical heterogeneity, and software package(s) used.	§3.7: no meta-analysis was performed, with the rationale given (heterogeneous measures, predominance of self-report, and too few comparable effect estimates). Synthesis is a structured narrative organised by design-based evidence stratum (§3.7, §4.5). No necessity or sufficiency parameters are reported, for the reason given in the Table 3 note.
Synthesis methods	13e	Describe any methods used to explore possible causes of heterogeneity among study results (e.g., subgroup analysis, meta-regression).	§4.5: conditions (locus of the meta-level operation, explicit metacognitive scaffolding, guidance, duration) and study characteristics (skill domain) were examined against direction of evidence within the contrast-based stratum.
Synthesis methods	13f	Describe any sensitivity analyses conducted to assess robustness of the synthesised results.	§4.6: the contrast-based pattern is recomputed after excluding the nine contrast-based studies that failed the final MMAT criterion (14 studies with a directional result, 79% positive against 74% for the full set).

Reporting bias assessment	14	Describe any methods used to assess risk of bias due to missing results in a synthesis (arising from reporting biases).	§3.7: not performed, with the reason stated (no common effect metric for funnel-plot or regression-based methods); the expected over-representation of published, positively framed findings is treated qualitatively in §5.4 and §5.8.
Certainty assessment	15	Describe any methods used to assess certainty (or confidence) in the body of evidence for an outcome.	§3.7: GRADE was not applied, with the reason stated (it presupposes a synthesised effect estimate). The design-based evidence strata (S1–S4, §3.7) reported together with the criterion-level MMAT profile (§4.6, Table A1) carry this function. The strata concern strength of inference about AI-related directional differences and are not a ranking of study quality.
**RESULTS**			
Study selection	16a	Describe the results of the search and selection process, from the number of records identified in the search to the number of studies included in the review, ideally using a flow diagram.	§3.4 and Figure 1 (PRISMA 2020 flow diagram): 3,807 records identified; 1,042 duplicates removed; 2,765 screened after de-duplication; 2,581 excluded by scripted triage; 184 candidate records; 161 within the review window; 86 excluded at title-and-abstract screening (33 without substantive metacognition measurement; 32 without AI mediation); 75 retained; 72 sought for retrieval after removing 3 conference papers; 71 full texts obtained; 13 excluded at full-text assessment; the 2 studies recovered at recall validation were added at full-text assessment, for 60 studies included.
Study selection	16b	Cite studies that might appear to meet the inclusion criteria, but which were excluded, and explain why they were excluded.	§3.4 (13 full-text exclusions by reason) and §5.8 (a known-item check: Zhan & Yan, 2025, which meets the substantive criteria but carries no L2 descriptor in title, abstract or keywords and so fell outside the language module).
Study characteristics	17	Cite each included study and present its characteristics.	Table B1 (all 60 studies with year, era, journal, AI application, functional orientation, direction and evidence stratum); reference-list entries for included studies are marked with an asterisk; full study-level codings in Supplementary S4 and S5.
Risk of bias in studies	18	Present assessments of risk of bias for each included study.	Table A1 (criterion-level MMAT grid for all 60 studies, with no aggregate score computed); interpretation in §4.6.
Results of individual studies	19	For all outcomes, present, for each study, summary statistics for each group (where appropriate) and an effect estimate and its precision (e.g., confidence interval), ideally using structured tables or plots.	Not applicable in the form specified: no effect estimates were extracted. Study-level direction of evidence and evidence stratum are presented for every study in Table B1, with the supporting codings in Supplementary S4–S5.
Results of syntheses	20a	For each synthesis, briefly summarise the characteristics and risk of bias among contributing studies.	§4.1 (characteristics), §4.6 (MMAT profile of the corpus), and §4.4–§4.5 (composition of the contrast-based stratum contributing to the directional analysis and to the descriptive cross-tabulation of conditions).
Results of syntheses	20b	Present results of all statistical syntheses conducted. If meta-analysis was done, present for each the summary estimate and its precision (e.g., confidence interval) and measures of statistical heterogeneity. If comparing groups, describe the direction of the effect.	§4.2–§4.5, Tables 2–3, Figures 4–9. No meta-analytic summary estimate was computed; direction of evidence is reported by stratum (Table 2) and by instructional condition (Table 3); no necessity or sufficiency parameters are reported.
Results of syntheses	20c	Present results of all investigations of possible causes of heterogeneity among study results.	§4.5 (conditions examined against direction) and §5.6 (interpretation against the pre-AI L2 metacognition literature).

Results of syntheses	20d	Present results of all sensitivity analyses conducted to assess the robustness of the synthesised results.	§4.6 (sensitivity of the contrast-based pattern to excluding the contrast-based studies that failed the final MMAT criterion).
Reporting biases	21	Present assessments of risk of bias due to missing results (arising from reporting biases) for each synthesis assessed.	Not assessed quantitatively (§3.7); discussed qualitatively in §5.4 and §5.8.
Certainty of evidence	22	Present assessments of certainty (or confidence) in the body of evidence for each outcome assessed.	§4.4 and §4.6 (direction by evidence stratum, and MMAT profile); §5.4 (the positive skew rests disproportionately on weaker designs).
**DISCUSSION**			
Discussion	23a	Provide a general interpretation of the results in the context of other evidence.	§5.1–§5.6, with §5.6 setting the corpus against the pre-AI L2 metacognition literature reviewed in §2.
Discussion	23b	Discuss any limitations of the evidence included in the review.	§5.4 (design limitations of the included evidence), §5.5 (construct-level limitations), §4.6 (methodological quality).
Discussion	23c	Discuss any limitations of the review processes used.	§5.8 (databases and specialist indexes not searched; scripted triage; known-item check; geographic and sector concentration; era base; incomplete final year).
Discussion	23d	Discuss implications of the results for practice, policy and future research.	§5.7 (researchers, teachers, tool designers, journals and reviewers) and §6.
**OTHER INFORMATION**			
Registration and protocol	24a	Provide registration information for the review, including register name and registration number, or state that the review was not registered.	§3.1: the review was not registered; the reason is given (PROSPERO does not accept reviews without a health-related outcome and no equivalent register was available).
Registration and protocol	24b	Indicate where the review protocol can be accessed, or state that a protocol was not prepared.	§3.1 and Supplementary S2 (protocol written before screening).
Registration and protocol	24c	Describe and explain any amendments to information provided at registration or in the protocol.	§3.1 and Supplementary S2, which contains a dated amendment log with fourteen entries. The amendments are of three kinds: changes to information sources, review window, search-string composition, document types and the number of research questions, all decided before screening, between the initial search (June 2026) and the CrossRef update search (7 August 2026); adjudication-derived coding rules and six added coding dimensions, decided during coding; and five amendments made during revision, namely the evidence-provenance rule for the regulatory-locus dimension, the addition of two studies recovered by recall validation, the replacement of the necessity analysis by a descriptive cross-tabulation, and the two successive full-text re-inspections of the contrast-based stratum (amendments 13 and 14).
Support	25	Describe sources of financial or non-financial support for the review, and the role of the funders or sponsors in the review.	Funding statement (end of manuscript).
Competing interests	26	Declare any competing interests of review authors.	Conflicts of Interest statement (end of the submitted manuscript).
Availability of data, code and other materials	27	Report which of the following are publicly available and where they can be found: template data collection forms; data extracted from included studies; data used for all analyses; analytic code; any other materials	Data Availability Statement, and Supplementary Materials S1–S6: search strategies (S1), protocol and amendment log (S2), this checklist (S3), the full coding table for the eight protocol dimensions (S4), the coding table for the dimensions added after the protocol with verbatim design-level evidence (S5),

		used in the review.	and the search-recall validation workbook (S6).

Table S3.2 PRISMA 2020 for Abstracts checklist

Section and Topic	Item #	Checklist item	Reported?
**TITLE**			
Title	1	Identify the report as a systematic review.	Yes — "A Systematic Review (2016–2026)" appears in the title.
**BACKGROUND**			
Objectives	2	Provide an explicit statement of the main objective(s) or question(s) the review addresses.	Yes — sentence 1 states that how the field studies metacognition under AI mediation has not been examined.
**METHODS**			
Eligibility criteria	3	Specify the inclusion and exclusion criteria for the review.	Partially — the abstract states the construct, the domain and the window (60 studies, 2016–May 2026); full criteria are in §3.3 for reasons of abstract length.
Information sources	4	Specify the information sources (e.g., databases, registers) used to identify studies and the date when each was last searched.	No — omitted for length; reported in §3.2 (six databases on three platforms, searched June 2026 and updated by a CrossRef-based supplementary search on 7 August 2026).
Risk of bias	5	Specify the methods used to assess risk of bias in the included studies.	Yes — "appraised with the Mixed Methods Appraisal Tool".
Synthesis of results	6	Specify the methods used to present and synthesise results.	Yes — coded on fourteen taxonomy-anchored dimensions; results presented as counts by direction and condition.
**RESULTS**			
Included studies	7	Give the total number of included studies and participants and summarise relevant characteristics of studies.	Partially — 60 studies and the era split are given; participant totals are not, because the corpus mixes designs for which a pooled participant count would be uninterpretable.
Synthesis of results	8	Present results for main outcomes, preferably indicating the number of included studies and participants for each. If meta-analysis was done, report the summary estimate and confidence/credible interval. If comparing groups, indicate the direction of the effect. If appropriate, consider the direction of the effect on important harms.	Yes — the three findings are reported with study counts (24 contrast-based studies; 98% and 66% of post-ChatGPT studies; 41% combining two or more orientations; one accuracy study in sixty; eight post-ChatGPT studies combining a contrast design with process data).
**DISCUSSION**			
Limitations of evidence	9	Provide a brief summary of the limitations of the evidence included in the review (e.g., study risk of bias, inconsistency and imprecision).	Yes — "that skew rests disproportionately on correlational and qualitative designs".
Interpretation	10	Provide a general interpretation of the results and important implications.	Yes — final sentence (a calibration- and process-data-anchored research agenda).
**OTHER**			
Funding	11	Specify the primary source of funding for the review.	No — MDPI places the funding statement in the article front matter rather than the abstract.
Registration	12	Provide the register name and registration number.	No — the review was not registered; stated in §3.1.

Registration and protocol statement

Registration. This review was not registered. PROSPERO accepts only reviews with a health-related outcome, and no equivalent register was available for reviews in educational technology or applied linguistics at the time the review began.

Protocol. A protocol was written before screening began and is provided as Supplementary S2. It specifies the review question, the eligibility criteria, the search strategy, the coding scheme for dimensions D1–D8, and the synthesis plan.

Amendments. All amendments are logged with dates in Supplementary S2. Of the fourteen logged amendments, five—information sources, the review window, the search-string composition, document types and the number of research questions—were decided before screening, between the initial search (June 2026) and the CrossRef update search (7 August 2026); adjudication-derived coding rules (including the boundary between metacognition proper and generic self-regulated learning, and the addition of a fifth AI-application category) and the six added dimensions D9–D14 followed during coding; and five amendments were made during revision in response to review: the evidence-provenance rule for the regulatory-locus dimension, the addition of two studies recovered by recall validation, the replacement of the necessity analysis by a descriptive cross-tabulation, and the two successive full-text re-inspections of the contrast-based stratum (amendments 13 and 14). Dimensions D9–D14 are documented in Table 1 and Supplementary S5.
